# Recent advances in application of hydrogel-based nanomaterials in breast cancer: from drug delivery, immunotherapy mechanisms to clinical applications

**DOI:** 10.1186/s12951-026-04238-z

**Published:** 2026-03-19

**Authors:** Yuanbing Xu, Dai Pan, Qinlian Yang, Chuyun Huang, Jing Zhou, Jun Wang, Qiuyun Li

**Affiliations:** 1 Department of Breast Surgery, Guangxi Medical University Cancer Hospital, Nanning, 530021 Guangxi China; 2 Department of Ultrasound, Guangxi Hospital Division of The First Affiliated Hospital, Sun Yat-Sen University, Nanning, 530021 Guangxi China; 3 Department of Physiology, School of Basic Medical Sciences, Guangxi Medical University, Nanning, 530021 Guangxi China; 4 Department of Clinical Laboratory Medicine, Southwest Hospital, Army Medical University, Chongqing, 400038 China

**Keywords:** Hydrogel, Breast cancer, Drug delivery, Immunotherapy, Stimuli-response, Breast reconstruction

## Abstract

Breast cancer remains one of the most prevalent malignant tumors affecting women worldwide and continues posing a major threat to global health. Current clinical treatments include surgery, chemotherapy, radiotherapy, targeted therapy, and endocrine therapy. However, these strategies are frequently limited by challenges such as drug resistance, elevated toxicity, adverse effects, and inadequate modulation of the tumor microenvironment (TME). Recent developments in nanotechnology have enabled the application of nanomaterial-based drug delivery systems that significantly improve delivery efficiency and biocompatibility, reduce drug toxicity and side effects, and demonstrate potential anticancer effects by modulating the TME. Hydrogels, a class of drug carriers, are characterized by a three-dimensional polymer network with high water absorption and retention capacity. Owing to their favorable biocompatibility, degradability, tissue-like physical properties, environmental responsiveness, and functional flexibility, hydrogels have been extensively utilized in biomedical applications, including bone regeneration, wound healing, antibacterial treatments, biosensing, and tumor therapy. Despite these advantages, hydrogels and nanomaterials still confront significant challenges when applied in breast cancer therapy. The integration of functional nanomaterials into the hydrogel matrix can form a novel multifunctional system. This transformation allows hydrogels to serve as targeted delivery platforms for anticancer nanodrugs, enabling synergistic therapeutic effects. This systematic review summarizes recent advances in hydrogel-based nanomaterials for breast cancer therapy, with emphasis on design strategies, mechanisms of action, and immunomodulatory applications. It also critically discusses current limitations and prospects of hydrogel-based nanomaterials. The objective of this review is to help lower interdisciplinary barriers and accelerate the clinical translation of hydrogel-based technologies toward safer, more personalized breast cancer treatments.

**Graphical Abstract:**

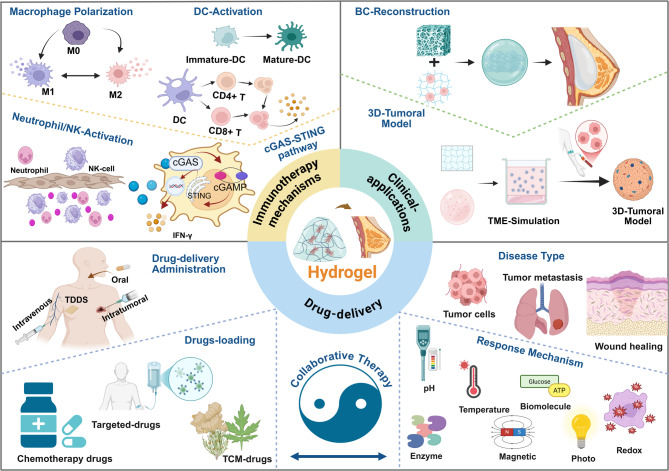

## Introduction

Breast Cancer (BC) is the leading malignant tumor in terms of incidence and mortality among women globally, with approximately 2.3 million new cases annually, representing 23.8% of all female cancer cases [1]. Notably, the incidence of BC in developing countries continues to rise [2]. Despite rapid advances in the diagnosis and treatments of BC, tumor recurrence and metastasis are still the main causes of death from breast malignant tumors worldwide [3, 4]. Traditional treatments, including chemotherapy, radiotherapy, and endocrine therapy, are frequently linked to systemic toxicity [5], immunosuppression [6], and radiation-induced damage [7, 8], which constrain the effectiveness and seriously affect patient quality of life. Consequently, there is an urgent need to develop novel therapeutic strategies with precise targeting, localized efficacy, and manageable side effects. In recent years, enormous progress has been made in cancer nanotechnology. Nanomaterials possess a high specific surface area, adjustable physicochemical properties, and excellent biocompatibility, which can facilitate targeted drug administration, enhance drug concentration in tumor tissues, and minimize harm to normal tissues [9, 10]. With the rapid advancement of nanotechnology, a diverse range of nanomaterials has been developed for tumor imaging and treatment, including metal NPs, polymer NPs, extracellular vesicles/cell membrane vesicles, etc. [11] (Table 1). Among these nanomaterials, hydrogel-based nanomaterials have gradually become a new research direction. Hydrogels are three-dimensional (3D) networks of hydrophilic polymers that can absorb large amounts of water. Owing to biocompatibility, degradability, physical similarity with tissues, environmental responsiveness, and functional adjustability, hydrogels have been widely used in biomedical applications, such as bone tissue regeneration [12, 13], wound healing [14, 15], antibacterial [16], biosensing [17], and tumor therapy [18].

**Table 1:** The advantages and disadvantages of different types of nanoparticles

NPs	Advantages	Disadvantages
Hydrogel	1. High biocompatibility and hydrophilicity; 2. High loading; 3. Excellent stimulus responsiveness; 4. Good colloidal stability and modifiability; 5. Injectability and deformability;	1. Synthesis complexity and quality control; 2. Relatively low mechanical strength;
Metal NPs	1. Unique physical properties (photothermal, magnetic, fluorescent, catalytic); 2. High mechanical strength and stability; 3. Easy to surface functionalization; 4. Excellent imaging contrast;	1. Potential long-term toxicity and non-degradability; 2. Possible leakage of heavy metal ions; 3. Difficulty in clearing the body (long-term accumulation);
Polymer NPs	1. Good biocompatibility and degradability; 2. Material designability and diversity; 3. Easy to Surface functionalization; 4. Multifunctional integration and stimulus responsiveness	1. Residual organic solvents; 2. Stability and storage challenges;
Extracellular vesicles/cell membrane vesicles	1. Natural biocompatibility and low immunogenicity; 2. Natural targeting and long circulation ability; 3. Cross biological barriers; 4. Contains natural bioactive substances;	1. Difficulty in separation and purification, with extremely low yield; 2. Low drug loading efficiency and complex methods; 4. Potential risk of transmission of carcinogens;

Given the unique physicochemical properties and broad application range of hydrogel-based nanomaterials, improving targeting efficiency is crucial for their biomedical applications. The strategies for improving targeting efficiency of hydrogel-based nanoparticle are mainly in the following four aspects: (1) Active targeting: Modify tumor specific recognition ligands on the surface of hydrogel-based nanomaterials to achieve "precise navigation"; (2) Passive targeting (EPR effect): Enhancing passive accumulation of tumor tissue by regulating the size, charge, and surface properties of hydrogel-based nanomaterials; (3) Tumor microenvironment (TME) response: Design an "on-demand release" system utilizing the characteristics of TME (acidity, high GSH, specific enzymes, etc.); (4) External regulation: precise activation of external stimuli; Realize precise spatiotemporal control through external physical fields (light, ultrasound, magnetic field, electricity, etc.). By integrating multifaceted targeting strategies, hydrogel-based nanomaterials exhibit enhanced therapeutic efficacy and reduced systemic side effects, thereby advancing the field of cancer nanomedicine towards greater precision and effectiveness.

Currently, research on hydrogels in the field of BC primarily concentrates on four key areas: (1) Drug delivery system: Hydrogels, as intelligent carriers, can achieve targeted and controlled drug release by responding to specific TME signals, significantly improving chemotherapy drug accumulation efficiency at the primary site and reducing systemic toxicity. (2) Construction of biological model: A 3D model of hydrogel was created in vitro by simulating the physical and chemical properties (such as stiffness and porosity) and biomolecular composition of tumor extracellular matrix (ECM) to replicate the heterogeneity of the BC microenvironment and provide a biomimetic platform for studying metastasis mechanisms and drug screening. (3) Synergistic therapy: Hydrogel-based drug delivery systems can integrate multiple therapeutic modalities, including chemotherapy, photothermal/photodynamic therapy (PTT/PDT), and immunotherapy, to overcome the limitations of single-agent treatments, such as drug resistance and adverse effects. The design objective is to balance the stability and synergistic efficacy of these multi-component systems, while addressing the challenges of energy and drug delivery to deep tumor tissues. (4) Breast reconstruction: Tissue repair reconstruction involves the use of hydrogels, which can be administered as injectable solutions or pre-formed scaffolds. These hydrogels play a crucial role in providing mechanical support for the regenerating tissue. Additionally, they promote vascularization and adipose tissue regeneration by incorporating bioactive agents.

**Scheme 1:**
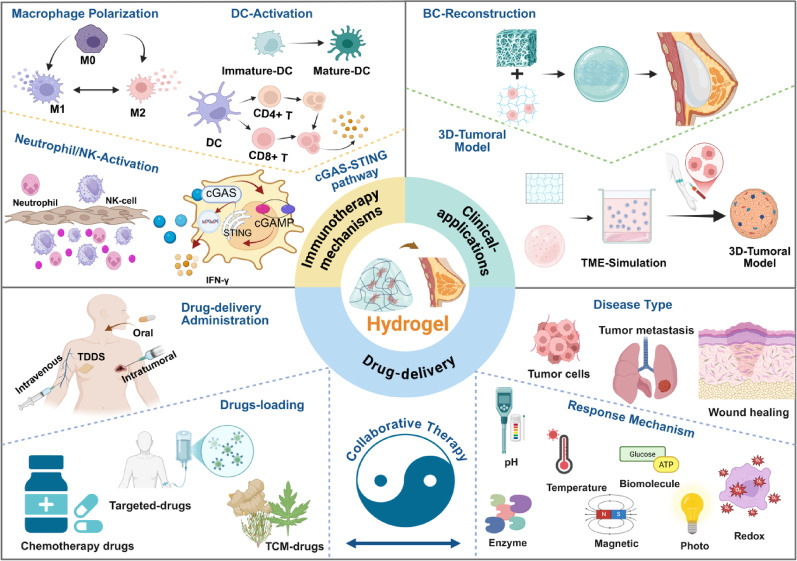
Overview of the application of hydrogel-based nanomaterials in breast cancer therapy

In this article, we review the research on hydrogel-based nanomaterials in BC treatment (Scheme 1) and discuss the challenges and opportunities in clinical application. The aim is to create an interdisciplinary knowledge map for researchers in the field, while also accelerating and promoting the development of the next generation of intelligent hydrogel theragnostic platforms.

## Hydrogel-based drug delivery systems for breast cancer therapy

### Drug loading strategies

BC therapy faces persistent challenges in balancing systemic drug exposure with localized therapeutic efficacy, frequently constrained by off-target toxicity and rapid clearance of conventional drug formulations [19, 20]. Nanomedicines are emerging as promising therapeutic agents for BC, addressing drug limitations and enabling precise delivery to primary and metastatic tumor sites through nanocarriers [21]. Hydrogels, serving as adaptable 3D structures, enable targeted drug delivery by encapsulating hydrophilic and hydrophobic compounds while regulating release dynamics [22, 23]. The transformation in BC treatment has been driven by continuous improvements in pharmacological therapies, with specific drugs addressing certain oncogenic weaknesses. Currently, the hydrogel-encapsulated drugs have been widely used for the treatment of BC, which can be primarily classified into three distinct categories. (1) Cytotoxic chemotherapeutics, as exemplified by doxorubicin (DOX, a DNA topoisomerase IIα inhibitor) [24] and paclitaxel (PTX, a microtubule-stabilizing agent) [25], remain fundamental in the current therapeutic framework by inducing mitotic catastrophe and apoptosis in rapidly proliferating tumor cells. (2) Targeted agents, particularly trastuzumab (HER2-targeted monoclonal antibody) [26] and abemaciclib (CDK4/6 inhibitor) [27], are revolutionizing the mode of treatment via precision blockade of oncogenic signaling cascades, thereby achieving unprecedented survival benefit in specific molecular subtypes. (3) Traditional Chinese medicine (TCM) compounds, including quercetin (a flavonoid derivative) [28, 29] and β-elemene (a sesquiterpene) [30], are emerging as promising next-generation adjuvants. These compounds uniquely orchestrate multimodal therapeutic effects by targeting tumor metabolic reprogramming, including the suppression of the Warburg effect and myeloid cell-mediated immunosuppression. Notably, these compounds exhibit minimal systemic toxicity, coupled with high therapeutic potency and sustainable efficacy, as demonstrated in preclinical-to-translational study models. In this section, we will introduce hydrogels loaded with different types of drugs for BC therapy.

#### Chemotherapeutic agents

##### Doxorubicin (DOX)

DOX, as a broad-spectrum anthracycline chemotherapy drug, plays a core role in neoadjuvant chemotherapy and postoperative adjuvant therapy for BC [31]. However, the clinical application was severely limited by these side effects, including drug-related cardiotoxicity, low tissue permeability, and multidrug resistance [32, 33]. Nanocarriers based on hydrogels offer a targeted drug-delivery strategy that can selectively accumulate at the tumor site, thereby significantly reducing systemic exposure and cardiotoxicity. The nanoscale size and tunable surface properties of these nanocarriers enhance their ability to penetrate deep into tumors. Additionally, these nanocarriers can co-deliver chemosensitizers or target ligands to overcome multidrug resistance (MDR) pathways, further improving therapeutic efficacy.

Targeted drug delivery via peptide-based self-assembled hydrogels can enhance the efficacy of chemotherapy and mitigate BC recurrence. Qi et al. [34] reported the co-assembly of a custom-designed hexapeptide and the chemotherapeutic agent doxorubicin, forming a hydrogel nanocarrier. This system enabled localized and sustained DOX release, significantly reducing the postoperative primary tumor recurrence rate (from 67 to 23%) and the lung metastasis rate (from 83 to 25%), without eliciting systemic toxicity. The delivery efficiency of target drugs can be enhanced by modifying the carrier platform. For instance, alginate (ALG) offers a versatile platform for drug delivery in various formulations, enabling optimized targeted delivery of DOX. Yang et al. [35] developed a multifunctional nanoparticle-loaded injectable ALG hydrogel that can effectively "loosen" tumor tissue via hyaluronidase-mediated degradation, thereby enhancing local DOX penetration at the tumor site. This approach can reduce non-specific toxicity and improve anticancer efficacy. Similarly, Jafari et al. [36] developed a p-selectin-targeting delivery system by covalently binding DOX to fucoidan, achieving controlled size distribution and sustained release. In vitro, fucoidan-DOX nanoparticles demonstrated active targeting of p-selectin, leading to increased cellular uptake and cytotoxicity against the MDA-MB-231 cell line. These innovative strategies show potential for treating triple-negative breast cancer (TNBC).

##### Paclitaxel (PTX)

Taxane-based chemotherapeutics, such as PTX and docetaxel (DOC), represent another classical treatment for BC. However, these agents often face challenges, including poor aqueous solubility and substantial adverse effects. Enhancing the targeted delivery of these pharmaceuticals while mitigating their toxicity has emerged as a prominent research focus. Wang et al. [37] developed a PTX-loaded bovine serum albumin (BSA) nanoparticle hydrogel (PTX@BN) by reacting BSA amine groups with 4-arm polyethylene glycol (PEG) terminated with phthalaldehyde. The PTX@BN hydrogel demonstrated prolonged PTX release over 30 days and effectively inhibited 4T1 BC cells in vitro. Compared to free PTX, the PTX@BN hydrogel significantly enhanced anticancer activity and extended survival time with minimal systemic toxicity. Palange et al. [38] engineered a deformable and degradable PEG-DTT micro-combined hydrogel particles (μCGP, diameter≈2 μm) encapsulating PTX-PLGA nanoparticles (≈200 nm). These μCGPs leveraged the "mechanical sieving" effect of pulmonary capillaries to accumulate near tumor vessels, gradually releasing PTX-SPN and sustaining high drug concentrations in the tumor stroma. In a mouse model of advanced TNBC lung metastasis, administering a modest PTX dose of 2 mg/kg every 3 days extended median survival to 95 days in the μCGP group, 2.1 times longer than with free PTX (46.5 days). This approach also doubled PTX accumulation in lung tissue and significantly reduced liver and kidney toxicity. Kwon et al. [39] investigated the potential of PTX to induce immunogenic cell death (ICD) and developed an ATP-modified PLGA nanohydrogel (PTX@NPpD-ATP). This system combined PTX-induced tumor antigen release with ATP-mediated recruitment of dendritic cells (DCs). Following PTX-induced ICD, the surface ATP served as a stable "find-me" signal in TME, resisting CD39/CD73-mediated degradation for up to 72 h, and continuously attracts CD11c⁺CD86⁺ mature DCs, which activated CD8⁺ T cells. In the immunologically "hot" CT26 model, systemic administration of PTX@NPpD-ATP (20 mg/kg, every 3 days for 4 doses) resulted in a median survival of 39 days. When combined with the PD-1 antibody, 75% of mice achieved complete tumor regression and developed tumor-specific immune memory.

##### 5-fluorouracil (5-Fu) and other antimetabolites

Classic antimetabolite agents, such as 5-Fu, capecitabine, and gemcitabine hold significant promise for improving breast cancer therapy [42]. However, the clinical application is hampered by a short half-life, low bioavailability, or significant systemic toxicity. Nanocarrier-based strategies have been extensively explored to enhance the targeting efficiency and safety profile of the antimetabolites [43]. He et al. [44] developed an injectable hydrogel system co-loaded with 5-Fu and the photosensitizer precursor 5-aminolevulinic acid (ALA) for the combined chemotherapy and photodynamic therapy of breast cancer. This hydrogel exhibited excellent injectability, self-healing properties, and mechanical stability, while also enabling adaptive degradation and on-demand release within the TME. In the 4T1 breast cancer model, the combined therapy effectively inhibited tumor growth. Taleblou et al. [45] devised an orally deliverable nanocomposite hydrogel drug delivery system (NHDDS) utilizing polyvinyl alcohol (PVA) and montmorillonite (MMT) for controlled capecitabine release in breast cancer treatment. In vitro investigations revealed a non-Fickian drug-release mechanism, with MMT content inversely controlling the release rate. The capecitabine-loaded NHDDS enhanced anticancer efficacy in 4T1 murine breast cancer models, significantly inhibiting tumor growth and reducing metastasis in vivo. These findings highlighted its potential as a promising oral platform for improved chemotherapy while minimizing systemic toxicity. Fang et al. [46] developed an injectable hydrogel system by cross-linking thiolated chondroitin sulfate with amphiphilic methacrylate micelles of Pluronic F127. This system enabled precise drug loading and controlled release of chemotherapy drugs and immunotherapy agents. The hydrophobic indoleamine 2,3-dioxygenase inhibitor D-1-methyltryptophan (D-1MT) was encapsulated within the micelle core, while the hydrophilic chemotherapy drug gemcitabine (Gem) was uniformly distributed throughout the hydrogel network. In a 4T1 tumor-bearing mouse model, the composite hydrogel effectively enhanced the anti-tumor immune response and reduced tumor lung metastasis by integrating chemotherapy and immunotherapy.

#### Targeted therapeutic agents

##### Trastuzumab

As a humanized monoclonal antibody, trastuzumab exerts therapeutic effects by binding to the HER-2 receptor, thereby inhibiting tumor cell growth and proliferation. Nevertheless, the clinical application of trastuzumab is limited by side effects such as drug resistance and cardiotoxicity.

**Fig. 1:**
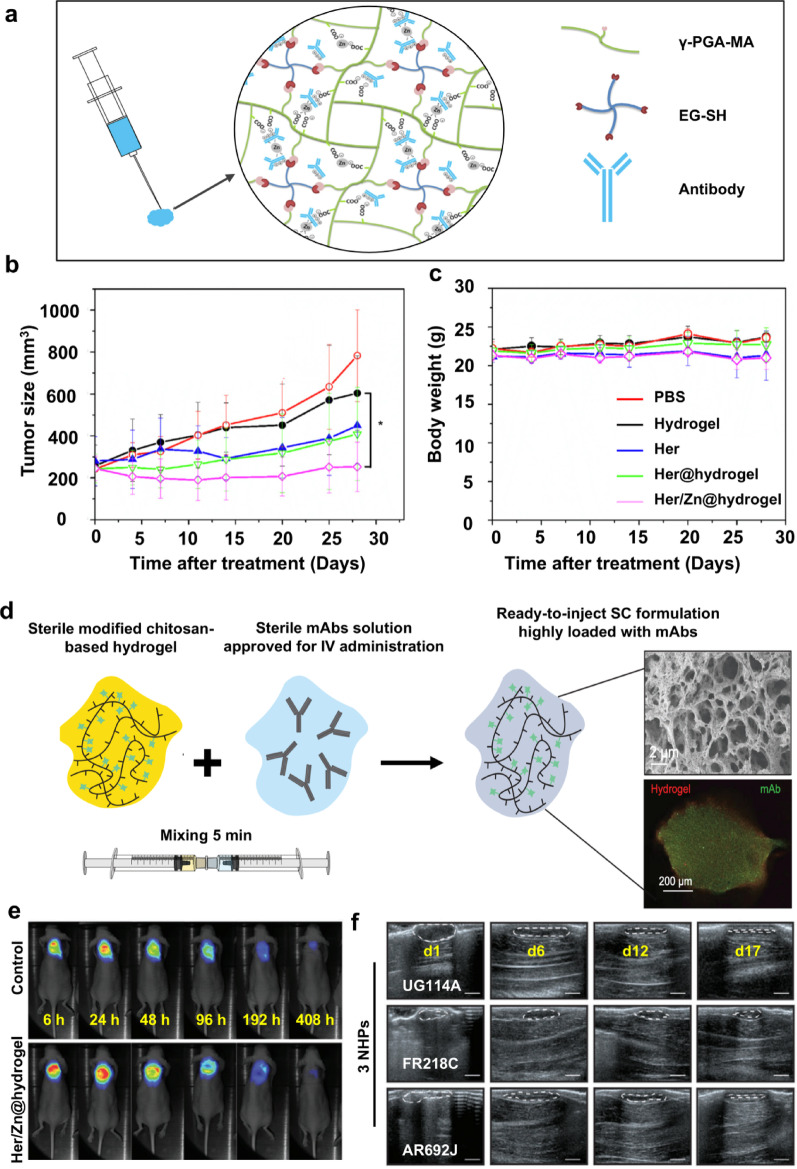
Schematic illustration of a trastuzumab-loaded hydrogel composed of c-poly glutamic acid (PGA)-maleimide (MA) and thiol end-functionalized 4-arm poly (ethylene glycol) (4-arm PEG-SH) (**a**); the changes of tumor volume (**b**) and body weight (**c**) in BT474-tumor bearing mice after various treatments. (Adapted with permission from [40]. Copyright^©^2019, Elsevier). The synthesis and characterization of the chitosan-based hydrogel loaded with mAbs (**d**); representative fluorescence images of the hydrogel and Her/Zn@hydrogel at different time points (**e**); representative US imaging depicting the degradation of the hydrogel at the injection site in the 3 NHPs. Scale bar: 1 cm (**f**). (Adapted with permission from [41]. Copyright^©^2023 Wiley–VCH GmbH)

In recent years, innovative sustained-release formulations have garnered significant attention as a means to deliver therapeutic antibodies. Nonetheless, developing sustained-release preparations with high drug loading remains a formidable challenge in practical applications. Lo et al. [40] successfully developed a high-loading, injectable, and sustained-release hydrogel for the subcutaneous delivery of trastuzumab (Fig. 1a). This Her/Zn@hydrogel system can achieve sustained drug release at a low rate (< 13%) over 4–7 weeks in vitro, and maintain a steady blood drug concentration (C_max_ of 279 μg/mL) at a significantly reduced level in vivo for 2–10 days, thereby achieving long-lasting and safe trastuzumab exposure. In the BT-474 xenograft model, Her/Zn@hydrogel produced 97.8% tumor growth inhibition over 4 weeks, which was significantly superior to the solution formulation (Fig. 1b). In addition, there was no significant change in body weight in each treatment group (Fig. 1c), suggesting good biocompatibility. Subcutaneous injection of monoclonal antibodies (mAbs) improved treatment outcomes and patient compliance. The "Zn^2+^-antibody nanocomplex" strategy not only reduced the risk of premature release but also significantly enhanced the anticancer effect in vivo, providing a feasible paradigm for the subcutaneous long-term delivery of monoclonal antibodies and for local/systemic treatment of BC.

Grea et al. [41] introduced a cationic chitosan-chitosan@DOTAGA injectable hydrogel as an alternative to traditional PEG- or PLGA-based systems for the subcutaneous delivery of mAbs with a molecular weight greater than 150 kDa. This system was designed to replace the clinical use of recombinant human hyaluronidase (rHuPH20). The hydrogel precursor was mixed with the antibody solution in a syringe and administered subcutaneously via a 25 G needle, where it rapidly self-assembled into a micro-porous interconnected network under physiological pH and ionic strength, enabling efficient mAb encapsulation. Scanning electron microscopy (SEM) analysis revealed a well-organized, multi-level microstructure with closely interconnected pores in the hydrogel. Meanwhile, confocal imaging further verified the homogeneous dispersion of antibodies within the hydrogel matrix (Fig. 1d). Fluorescence imaging in mice demonstrated sustained trastuzumab release from the hydrogel system for at least 7 days, confirming the hydrogel's long-term in vivo release capacity (Fig. 1e). In 3 nonhuman primates (NHPs), high-frequency ultrasound imaging suggested even longer release times for Her/Zn@hydrogel (Fig. 1f). These findings indicated that hydrogel-based trastuzumab delivery can achieve favorable sustained release in vivo, providing promising evidence for clinical translation.

##### Immune Checkpoint Inhibitors (ICIs)

ICIs can activate systemic anticancer immunity by blocking immunosuppressive pathways, such as the PD-1/PD-L1 pathway. Injectable hydrogels enhance the precision of ICI delivery to the TME via local retention and slow-release mechanisms. This approach enhances therapeutic efficacy and reduces systemic exposure, thereby increasing the effectiveness and minimizing toxicity in targeted immunotherapy. Cheng et al. [47] designed an injectable silk fibroin hydrogel capable of co-delivering cGAMP nanoparticles (STING agonist), DOX (ICD inducer), and immunomodulators (anti-PD-1/OX40L). This combination enabled sustained local STING pathway activation and effective remodeling of the tumor immune microenvironment. This approach significantly inhibited the progression, metastasis, and recurrence of various immunologically "cold" tumors, such as TNBC, glioblastoma, hepatocellular carcinoma, and pancreatic ductal adenocarcinoma. Liu et al. [48] employed injectable supramolecular hydrogels for tumor local PD-L1 blockade and chemo/immunotherapy. An injectable supramolecular hydrogel (HDU) co-delivered DOX and the D-peptide antagonist DPPA-1, which achieved a synergistic anticancer effect. DOX induced ICD and released tumor antigens, while DPPA-1 blocked the PD-1/PD-L1 pathway and enhanced CD8⁺ T cell infiltration and IFN-γ/TNF-α secretion. This strategy offered new avenues for developing safer and more effective immunotherapy regimens.

##### Abemaciclib (Abe)

Studies have demonstrated that long-term use of ICIs can lead to upregulation of the immunosuppressive enzyme indoleamine 2,3-dioxygenase 1 (IDO-1), activate the tryptophan-kynurenine (Trp-Kyn) pathway, and promote the expansion of regulatory T cells (Tregs), thereby diminishing the efficacy of immunotherapy [49]. As an approved CDK4/6 inhibitor for HR⁺/HER2⁻ breast cancer, Abemaciclib not only targets and inhibits the tumor cell cycle but also effectively induces immunogenic cell death and activates the anti-tumor immune response. This property offers a novel strategy to tackle the clinical challenge of inadequate responses to ICIs in TNBC.

**Fig. 2:**
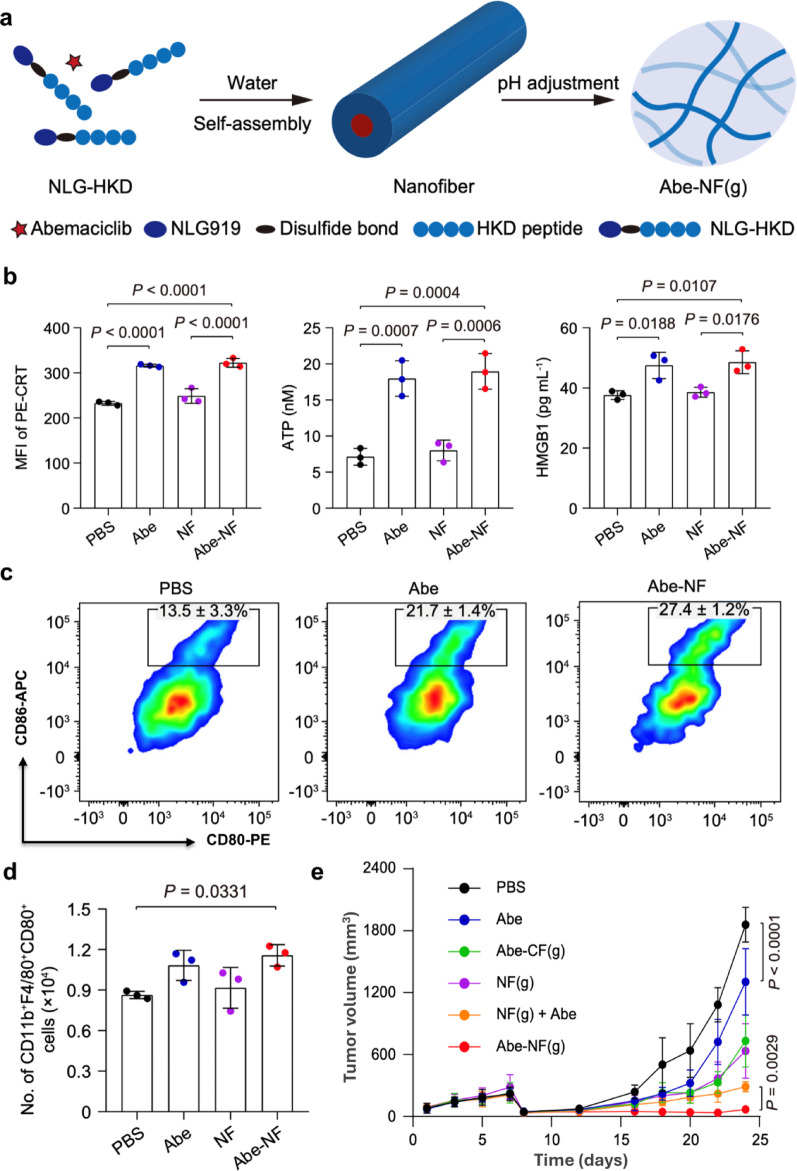
Schematic illustration of the design and mechanism of the injectable hydrogel as a neoadjuvant immunotherapy (**a**); assessment of ICD induction in MDA-MB-231 breast cancer cells 24 h after treatment (**b**). Representative flow cytometric images of DC maturation in vitro 24 h after co-culture with 4T1 cells under different treatments (**c**); the number of M1 in the coculture of BMDMs and pretreated 4T1 cells (**d**). The changes of tumor volume under various treatments (**e**). (Adapted with permission from [27]. Copyright^©^2025, Springer Nature)

Zhu et al. [27] developed an injectable supramolecular hydrogel (Abe-NF gel) composed of Abe and the prodrug of the IDO-1 inhibitor NLG919 (NLG-HKD) (Fig. 2a). Following intratumoral injection, the hydrogel continuously released Abe and NLG919 as a drug reservoir. Abe not only induced ICD (Fig. 2b) but also promoted DC maturation (Fig. 2c), macrophage polarization to an M1 phenotype (Fig. 2d), and enhanced IL-2 secretion by cytotoxic T lymphocytes. Notably, Abe can upregulate IDO-1 expression. Meanwhile, the simultaneously released NLG919 effectively inhibited its activity, thereby reversing the immunosuppressive microenvironment. In the 4T1-luc TNBC model, a single dose of Abe-NF gel as neoadjuvant immunotherapy significantly inhibited postoperative tumor recurrence (Fig. 2e), effectively reduced lung metastasis, and prolonged the survival period of mice. However, it is important to note that the therapeutic efficacy of this strategy was highly dependent on an intact adaptive immune system. Data from the NOD-SCID mouse model, which lacks functional T and B cells, clearly demonstrated that the anti-tumor efficacy of Abe-NF (g) was significantly diminished in the absence of a functional adaptive immune response. Therefore, the clinical translation of this promising neoadjuvant immunotherapy platform should carefully consider patient stratification based on immune profiles, and future investigations should evaluate its applicability across different breast cancer subtypes with varying TMEs.

#### Traditional Chinese medicine (TCM) and natural compounds

TCM offers a vast repository of therapeutic knowledge and drug resources accumulated over millennia. The discovery of artemisinin from Artemisia annua by Nobel laureate Tu Youyou and her team, which has saved millions of lives, exemplifies the remarkable potential of TCM in addressing major diseases. Notably, active compounds like Elemene (ELE) and Quercetin (QC), derived from Chinese herbal medicines, have emerged as promising anticancer agents with clinical approval.

##### Elemene

**Fig. 3:**
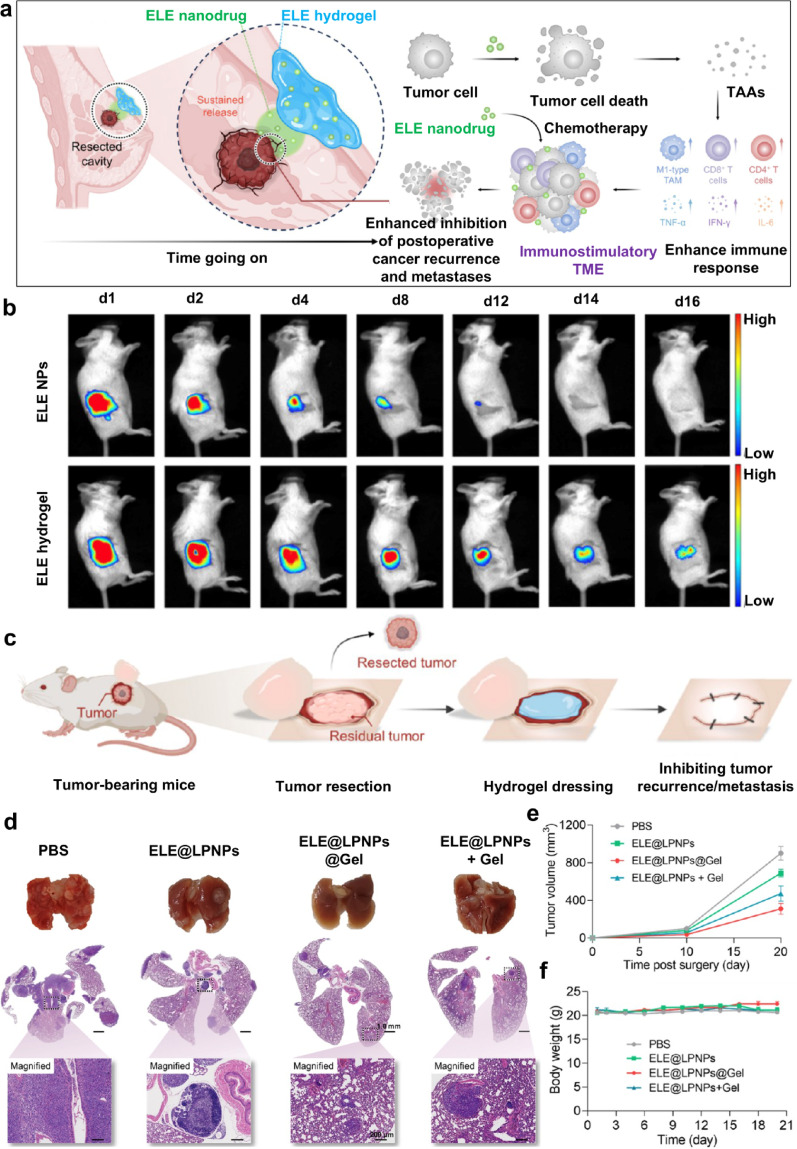
Schematic diagram of the anti-tumor immune mechanisms of ELE@LPNPs @Gel (**a**); Fluorescence imaging of ELE NPs and ELE hydrogel at different time points (**b**); Schematic illustration of the operation procedure of the hydrogel inhibiting tumor recurrence and metastasis (**c**); Photographs of lung tissue from mice and H&E stained images after various treatments (**d**); The changes of tumor volume (**e**) and body weight (**f**) of the mice under various treatments. (Adapted with permission from [30]. Copyright^©^2025, American Chemical Society)

Natural plant extracts are categorized into terpenoids, flavonoids, phenolic acids, and alkaloids according to the chemical structure and function of their secondary metabolites. ELE, a representative terpene, exhibits diverse biological activities, including anticancer, anti-inflammatory, antibacterial, antioxidant, antiviral, and immunomodulatory effects. Xian et al. [30] engineered an injectable dopamine-conjugated hyaluronic-acid hydrogel (HADA) that embedded ELE-loaded lipid–polymer hybrid nanoparticles (ELE@LPNPs) (Fig. 3a). This gel continuously released the drug for 11 days in vitro, with fluorescence detectable for 20 days post-implantation, significantly extending local drug retention (Fig. 3b). A 4T1-Luc BC postoperative model (Fig. 3c) revealed that local implantation of the sustained-release hydrogel significantly inhibited tumor recurrence, with a 96% inhibition rate, outperforming ELE@LPNP (65.5% inhibition rate). This strategy also effectively prevented distant lung metastasis (Fig. 3d) and recurrence of residual tumors (Fig. 3e). Importantly, the body weight of mice in all treatment groups remained stable, and no significant toxic side effects were observed (Fig. 3f). These findings highlighted the remarkable potential of elemene, an active ingredient of TCM, as a promising therapeutic approach for the management of BC following surgical intervention. In addition to enhancing immune function, the anticancer mechanisms of ELE include inducing cell apoptosis by blocking the cell cycle, regulating bioactive proteins, and mediating signaling pathways. β-Elemene has been shown to inhibit the proliferation of human BC MCF-7 cells by inducing G₁/S cell cycle arrest and promoting apoptosis [50]. Xie et al. [51] further corroborated that β-elemene can suppress TNBC proliferation by concurrently downregulating insulin-like growth factors and upregulating aging-related proteins. Additionally, β-elemene can enhance the chemotherapeutic efficacy of 5-fluorouracil against triple-negative BC, an effect potentiated by inhibition of the PI3K/AKT signaling pathway and synergistic modulation of the RAF-MEK-ERK signaling cascade [52].

##### Quercetin (QC)

QC, a flavonoid with antioxidant and chemopreventive properties, has been shown to inhibit BC cell growth [53, 54]. However, its application is restricted by low water solubility and poor permeability. To address this, Nematollahi et al. [28] developed a γ-Alumina-reinforced chitosan/polyvinylpyrrolidone (CS/PVP/γ-Alumina) nanocomposite loaded with QC using a double water-in-oil-in-water (W/O/W) emulsion method. In vitro, this nanocomposite reduced MCF-7 cell viability to 9% and induced apoptosis in 95.3% of cells, surpassing the efficacy of free QC and reported QC-based carriers. Similarly, Sabzini et al. [29] developed chitosan/halloysite nanotube/g-C_3_N_4_ (CS/HNT/g-C_3_N_4_) nanocarriers using a W/O/W method to load QC, achieving an encapsulation efficiency as high as 86%. The release study indicated that the release of QC was 76% in an acidic microenvironment (pH 5.4) over 96 h, with only 38% release at physiological pH. In vitro studies demonstrated that CS/HNT/g-C_3_N_4_/QC reduced MCF-7 cell viability by 2%, 35%, and 65% compared to free QC and other QC carriers, respectively. These findings highlighted the synergistic effects of nanoporous inorganic materials and natural polysaccharides in modulating the crystallization behavior and pH responsiveness of QC, leading to efficient loading, controlled release, and enhanced cytotoxicity against BC cells.

##### Other TCM-derived agents

In addition to ELE and QC, other natural anticancer compounds, such as curcumin (CUR), apigenin, and luteolin, also exhibit significant potential in anticancer treatment. These bioactive molecules have been extensively studied for their ability to modulate multiple oncogenic pathways, including cell proliferation, apoptosis, angiogenesis, and immune evasion, thereby offering synergistic or additive effects when combined with conventional chemotherapeutics. For example, to enhance water solubility and bioavailability of CUR, Li et al. [55] developed a thiolated chitosan-coated CUR liposomal hydrogel (CSSH/CUR-LIP gel) for prolonged local therapy following BC surgery. This system enhanced the aqueous solubility and stability of CUR by liposome encapsulation (CUR-LIP). Subsequently, the system transformed into a temperature-sensitive injectable hydrogel enveloped by CSSH, demonstrating a range of functionalities including in situ gel formation, controlled drug release, prevention of cancer recurrence, and facilitation of tissue regeneration. Apigenin, a natural flavonoid, exhibits broad-spectrum anticancer properties and has demonstrated significant anticancer effects across multiple cancers. Sudhakaran et al. [56] showed that the dietary flavonoid apigenin induced transcriptome-wide reprogramming of alternative splicing (AS) in TNBC cells. Apigenin specifically modulated AS events enriched in hnRNPA2 substrates, switching cancer-associated isoforms to non-tumor-like profiles. This splicing reprogramming favored pro-apoptotic and anti-proliferative isoforms, leading to increased apoptosis and reduced tumor growth in vitro and in vivo. Apigenin significantly decreased tumor volume in MDA-MB-231 xenograft mice by altering AS of key genes involved in cell death pathways, without affecting non-tumor cells. Wang et al. [57] developed a chitosan-based in situ sustained-release hydrogel system co-delivering the GLUT1 inhibitor apigenin and the chemo-drug gemcitabine for post-surgical cancer treatment. In CT26 and 4T1 tumor models, the combination therapy (apigenin-gemcitabine) effectively inhibited tumor recurrence and metastasis, induced robust apoptosis, and reshaped the tumor immune microenvironment by promoting M1 macrophage polarization and enhancing CD8^+^ T cell infiltration. Additionally, the combination therapy exhibited increased immune memory responses, reducing tumor rechallenge growth. These findings demonstrated that the apigenin-gemcitabine co-loaded hydrogel was a safe and effective strategy for preventing cancer recurrence and metastasis after surgery.

In this section, we have focused on categorizing the drugs commonly delivered via BC hydrogels, including chemotherapy drugs, targeted therapeutic agents, TCM, and natural compounds. Table 2 summarizes these drugs by loading strategy, outlining the advantages and disadvantages of each.

**Table 2:** Different drug-loading patterns of hydrogels for the treatment of BC

Drug-loading strategy	Classification	Drugs	Advantages	Disadvantages	Ref
**Single-drug delivery**	Chemotherapy	DOX	High drug loading	Cardiotoxicity risk	[34]
	Chemotherapy	PTX	Controlled release	High hydrophobicity	[58]
	Chemotherapy	DOC	Long circulating time	Larger particle sizes	[59]
	Chemotherapy	5-FU	Precise drug release	Hand-foot syndrome	[60]
	Chemotherapy	Mitoxantrone	Lymphatic tracer	Skin staining	[61]
	Chemotherapy	Cisplatin	Broad-spectrum anticancer drug	Nephrotoxicity and neurotoxicity	[62]
	Chemotherapy	Capecitabine	Better bioavailability and lower toxicity	Palmar-plantar erythrodysesthesia	[45]
	Targeted-therapy	Trastuzumab	Specifically target the HER2 receptor	Potential cardiotoxicity	[40]
	Targeted-therapy	ICIs	Enhance immune response	Immune-related adverse events	[47]
	Targeted-therapy	Abe	Inhibit the activity of CDK4/6	Diarrhea	[27]
	TCM	ELE	Anticancer activity	Limited solubility	[30]
	TCM	QC	Antioxidant activity	Photodegradation risk	[28]
	TCM	CUR	Anticancer potential	Rapid metabolism	[55]
	TCM	Apigenin	Better biocompatibility	Low bioavailability	[57]
**Dual-drug delivery**	Dual-Chemotherapy	DOX + DOC	Dual cell cycle disruption	Myelosuppression	[63]
	Dual-Chemotherapy	PTX + DOX	Microtubule disruption	Cardiotoxicity	[64]
	Dual-Chemotherapy	Cisplatin + PTX	Double DNA damage	Myelosuppression	[65]
	Dual-Chemotherapy	DOX + Gemcitabine	Synergistic s-cell death	Myelosuppression	[66]
	Dual-Chemotherapy	DOX + Cisplatin	Dual DNA damage mechanisms	Nephrotoxicity	[67]
	Chemotherapy + Immunotherapy	PTX + aPD-L1	Synergistic antitumor effect	Immune-related adverse events	[68]
	Chemotherapy + Immunotherapy	DOX + aPD-L1	Enhanced immune response	Cardiotoxic risk	[69]
	Chemotherapy + TCM	DOX + QC	Natural compound synergy	Herb-drug interactions	[70]
	Immunotherapy + TCM	CUR + Indoximod	Immune modulation enhancement	Bioavailability	[71]
	Immunotherapy + Adjuvants	aPD-L1 + CpG ODN	Immune activation	Off-target effects	[72]
	Chemotherapy + Analgesics	Esketamine + DDP	Pain control with chemotherapy	Neurotoxic risk	[73]
	Chemotherapy + nucleic acid drugs	DOX + siRNA	Gene-specific targeting	Off-target effects	[74]

### Stimuli-responsive drug release mechanisms

**Fig. 4:**
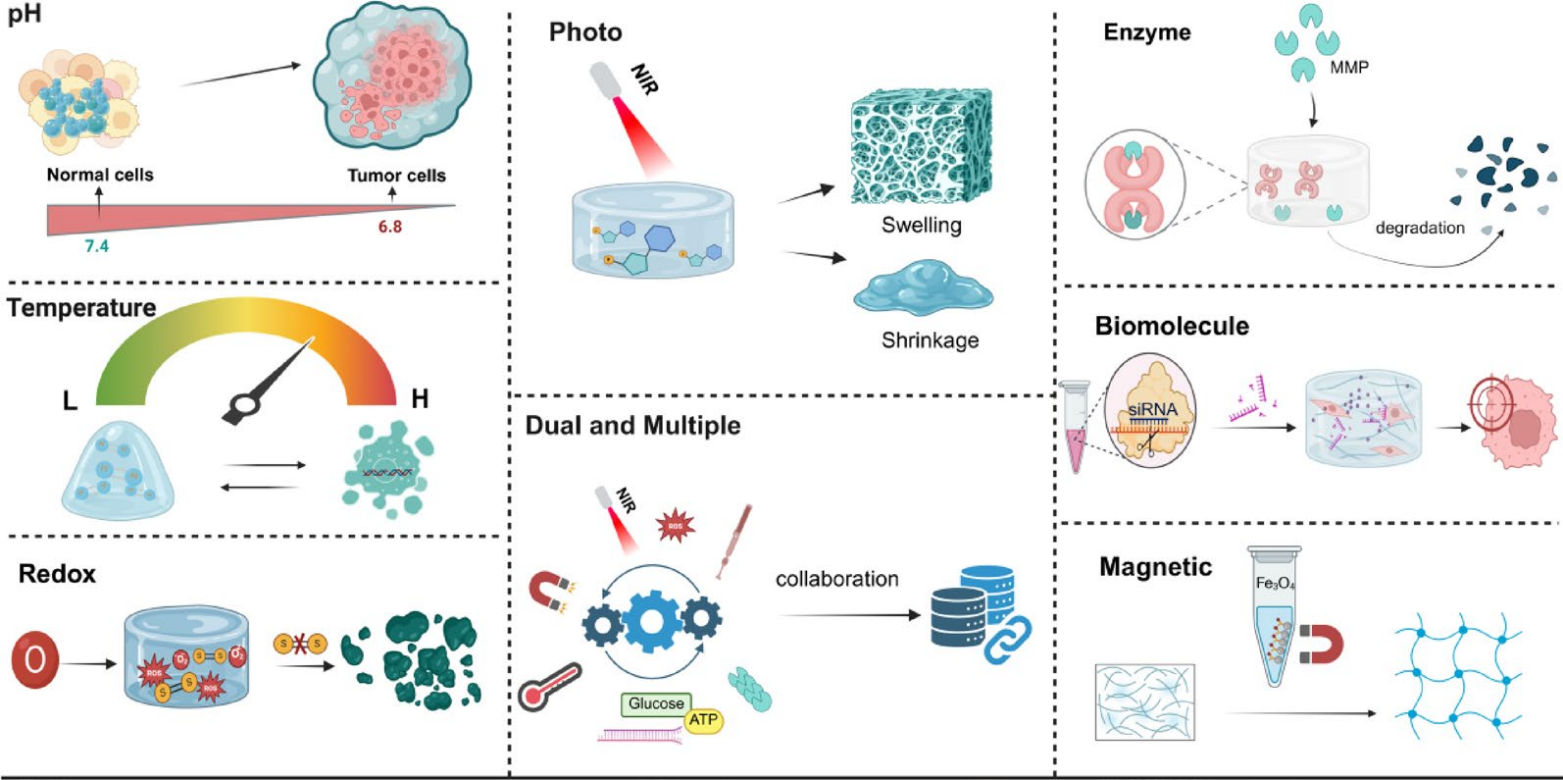
Brief mechanism diagram of different response strategy principles

Stimuli-responsive nanoplatforms capitalize on various internal and external triggers to achieve efficient and targeted drug delivery for cancer therapy. By enabling precise drug release at the tumor site, these systems enhance therapeutic efficacy while minimizing off-target side effects, representing a significant advancement over traditional chemotherapy [75]. Among these platforms, hydrogel-based nanomaterials stand out for their exceptional responsiveness to TME. As summarized in Fig. 4, hydrogels can be engineered to respond to a wide array of stimuli, including pH, temperature, redox conditions, enzymes, light, magnetism, and biomolecular signals, as well as their dual- or multiple-responsive combinations. The following sections will provide a comprehensive analysis of each stimulus–response modality and its application in breast cancer therapy.

#### Internally stimuli-responsive systems

The acidic pH, elevated H_2_O_2_ levels, and hypoxic conditions characteristic of TME can impede the efficacy of conventional chemotherapies. However, these TME features can also be leveraged for targeted drug delivery using responsive hydrogel systems. Hydrogels with complex network structures can be designed to autonomously initiate drug release mechanisms in response to specific TME cues. For instance, acidic pH can trigger the breakdown of Schiff base bonds, high H_2_O_2_ levels can promote the cleavage of oxide-sulfide bonds, and hypoxic conditions can accelerate drug release through reduction-sensitive bonds. This "internal response" strategy capitalizes on the tumor's intrinsic pathophysiological properties, enabling selective activation of therapeutic agents at the tumor site. In essence, the hydrogel system can recognize and respond to the unique "pathological code" dictated by the TME, promptly releasing the drug upon detection of these local signals, thereby enabling precise passive targeting.

##### pH-responsive

pH-responsive hydrogels are a class of smart materials that can selectively respond to the acidic conditions characteristic of TME. These hydrogels enable precise, controlled drug release within the TME, thereby enhancing therapeutic efficacy and minimizing damage to healthy tissues, offering new opportunities for tumor treatment. Zheng et al. [76] developed an injectable hydrogel composed of oxidized hyaluronic acid and carboxymethyl chitosan (CMCS/OHA-MET20). Metformin was covalently grafted to the hydrogel via dynamic imine bonds, enabling on-demand release triggered in the tumor acidic microenvironment. In an in vivo BC postoperative model, the hydrogel exhibited a cumulative drug release rate of 79.3% within 6 h at pH 5.5, nearly twice that observed under physiological pH conditions. The tumor recurrence rate in the CMCS/OHA-Met20 group decreased to 20% after 28 days compared with the free-drug group (60%). This strategy not only retained metformin's mitochondrial caspase-3/PARP apoptotic pathway activity but also reduced normal tissue toxicity by 30% through HA-CD44 targeting, providing a clinically viable, intelligent delivery platform for postoperative local chemotherapy. Cimen et al. [77] constructed an injectable, self-healing, pH-responsive Gelatin-PEG/Laponite composite hydrogel (Gel-ADH/diBA-PEG/LAP@DOX) that exhibited strong gel stability and prolonged drug release. The hydrogel released 2.4 times more DOX at pH 5.5 than at physiological pH 7.4 over 72 h, effectively inhibiting tumor cell growth and reducing systemic toxicity. This approach introduced a "one injection, sustained controlled release" model for local chemotherapy after surgery for solid tumors, which addressed the need for long-term, implantable drug systems in clinical settings.

##### Redox-responsive

The redox state in the TME not only affects the metabolism and growth of tumor cells but also plays a key role in tumor drug sensitivity and resistance. The increased metabolic activity of tumor cells is typically accompanied by increased oxidative stress, resulting in a higher intracellular concentration of L-Glutathione (GSH). For example, Zhuang et al. [78] designed a calcium ALG-Fe-MOF composite hydrogel that can be generated in situ in tumors to achieve synergistic iron death therapy for BC through multi-pathway amplification of redox imbalance. The hydrogel utilized a local high concentration of Ca^2+^ to trigger the transient crosslinking of ALG, thereby realizing precise implantation and continuous retention of Fe-MOF, PTX, and chlorin e6 (Ce6). Fe-MOF exhibited peroxidase and glutathione oxidase-like activities, and catalyzed the Fenton reaction to generate hydroxyl radicals (•OH) and deplete GSH in an acidic microenvironment. Low-dose PTX down-regulated SLC7A11 to block GSH synthesis further and reduce pH to enhance catalytic efficiency. Sonosensitizer Ce6 generated ^1^O_2_ under ultrasound excitation, superimposed reactive oxygen species (ROS) burst, and finally induced lipid peroxidation and GPX4 inactivation. In vivo experiments demonstrated that this strategy achieved a 90% tumor inhibition rate in the 4T1 BC model within 14 days, which was considerably better than that of the single component.

##### Enzyme-responsive

Hydrolytic enzymes, including matrix metalloproteinases and cathepsins, are prominently present in TME. Enzyme-responsive hydrogels leverage the elevated expression of tumor-specific enzymes to trigger drug release as needed. By identifying and cleaving specific enzyme sequences, these "signals" can trigger bond rupture, initiate drug release, alter nanostructure configurations, or disassemble the network, thereby enabling accurate spatial and temporal control of lesions. For example, Sun et al. [79] reported a one-component deformable peptide-photosensitizer conjugate (PPC) for "mild photothermal-immune" synergistic therapy of BC. The PPC is composed of a PD-L1 antagonist peptide (CVRARTR), matrix metalloproteinase-2 (MMP-2) enzyme cleavage sequence (PLGLAG), self-assembly motif (FFKYG), and photosensitizer Purpurin-18, which can self-assemble into 230 nm nanospheres in water. After intravenous injection, the overexpression of MMP-2 cleaved PPC at the tumor site, releasing antagonistic peptides that led to the co-assembly of residues into nanofibers, thereby enhancing drug release and retention. In vivo studies demonstrated that PPC nanospheres facilitated CTL infiltration and promoted DCs maturation under laser irradiation, thereby enhancing the sensitivity of 4T1 tumor cells to immune checkpoint blockade therapy. Consequently, PPC nanospheres effectively inhibited tumor growth locally and distally while preventing the development of lung metastases.

##### Biomolecule-responsive

Hydrogels serve as pivotal responsive drug delivery systems in breast cancer therapy, precisely targeting specific receptors and biomolecules within the TME, including ATP, glucose, and nucleic acids. ATP-responsive hydrogels exploit the elevated ATP levels in tumor cells to initiate drug release. The ligand-aptamer interaction alters the aptamer's conformation from a bound to an unbound state, a mechanism utilized in hydrogels crosslinked by ATP aptamers [80]. For instance, Chen et al. [81] developed a nano-metal–organic framework (nMOFs) containing Zr^4+^ ions and ATP nucleic acid ligand sequences. nMOFs can automatically unlock by forming ATP-receptor complexes in environments with high ATP levels, thereby enabling drug release. Glucose-responsive hydrogels can sense changes in glucose levels in TME. When glucose binds to specific receptors, it triggers the physical response of the hydrogel. Hao et al. [82] developed a combination of near-infrared (NIR) laser and glucose-responsive hydrogel for the treatment of breast cancer. In the presence of glucose and under NIR irradiation, this hydrogel generates highly reactive hydroxyl radicals (•OH) via a cascade reaction, inducing oxidative stress and cancer cell death. The DNA/RNA-mediated hydrogel system modulates endogenous microRNA (miRNA) expression in cancer tissues. To deliver the nucleoside analog fluorouridine, Zhang et al. [83] engineered spherical DNA and RNA nanogels loaded with fluorouridine via solid-phase synthesis or enzymatic transcription, replacing the nucleoside thymidine (T) in single-stranded nucleic acids. These nanogels showed precise drug loading, substantial cellular uptake, and effective induction of tumor cell apoptosis.

#### Externally triggered release systems

The external response mechanism actively regulates nanocarrier behavior in response to specific external stimuli (e.g., temperature, light, magnetic fields) to achieve precise drug release. Unlike mechanisms dependent on the TME, this mechanism controls the timing and location of drug delivery externally, thereby enhancing treatment accuracy and controllability.

##### Temperature-responsive

The thermal response mechanism utilizes temperature changes as triggering conditions. When nanocarriers are exposed to specific temperatures or physicochemical stimuli, they can undergo structural changes that facilitate drug release. For instance, some temperature-sensitive polymers, such as poly (N-isopropyl acrylamide) (PNIPAAm), undergo reversible phase transitions at specific critical temperatures. Below the lower critical solution temperature (LCST) of approximately 32 °C, PNIPAAm is hydrophilic and soluble, but above the LCST, it becomes hydrophobic and insoluble, thereby accelerating drug release. Qiao et al. [84] developed a temperature-controlled system to manipulate and monitor HER2 receptor clustering on cell membranes. This system enables the in-situ conformational transition of polymer-peptide conjugates, ultimately leading to effective inhibition of breast cancer cell proliferation. This temperature-triggered receptor cross-linking induced a 4.6-fold increase in HER2 intracellular tyrosine phosphorylation, which significantly blocked HER family heterodimerization. Consequently, the proportion of EdU-positive cells was reduced to 7.7%, achieving highly selective inhibition of HER2-positive breast cancer cell proliferation.

##### Photo-responsive

The photo-response mechanism activates the photosensitive components in the nanocarrier using specific wavelengths of light, like NIR, to induce structural changes or generate photothermal effects, leading to controllable drug release. As a non-invasive stimulus, light offers spatiotemporal control and precise tumor targeting, thereby minimizing its effects on surrounding healthy tissues. Lan et al. [85] designed a photo-controlled polyunsaturated fatty acid-doped liposomal hydrogel for flexible photoimmunotherapy. The liposomal hydrogel Lp (DHA)@CP Gel was loaded with the photosensitizer Ce6 and programmed death ligand 1 antibody (PD-L1), which can be repeatedly induced to release PD-L1 in response to PDT under light irradiation. The ICD effect of PDT transformed a "cold" breast tumor into a "hot" one, which then assisted in the cascade release of PD-L1, synergistically enhancing immunotherapy. Wu et al. [86] developed red blood cell membrane-coated nanoparticles (HDC-DM) loaded with photosensitizers to prevent lung metastasis from TNBC. In the 4T1 breast cancer model, NIR laser irradiation of the tumor site triggered the DiR photosensitizer to induce photothermal effects, leading to the rupture of erythrocyte membranes and subsequent drug release. The results showed that HDC-DM could significantly inhibit tumor growth and effectively prevent the formation of lung metastases.

##### Magnetic-responsive

The magnetic response mechanism exploits the behavior of magnetic nanoparticles under magnetic fields to direct the movement and aggregation of nanocarriers for targeted drug delivery and release. Magnetic nanoparticles, such as Fe_3_O_4_, can aggregate or vibrate in response to a magnetic field, leading to structural changes within the nanocarrier and thereby accelerating drug release. Additionally, the magnetothermal effect is frequently employed in these systems to heat magnetic nanoparticles, thereby activating thermosensitive drug carriers. Lu et al. [87] developed a low-frequency magnetic field-modulated tumor cell membrane camouflage vortex magnetic nanorings (DOX-VMAs@CM) for targeted therapy of TNBC, which enabled precise drug delivery and release under varying low-frequency magnetic fields. DOX-VMAs@CM significantly enhanced drug accumulation and penetration in tumor tissues by vibration of a low-frequency magnetic field in the 4T1 breast tumor-bearing mouse model, induced immunogenic cell death, and activated anti-tumor immune response. Similarly, Ji et al. [88] introduced an intermittent low-frequency rotating magnetic field (LF-RMF) approach to specifically disrupt F-actin in breast cancer cells, resulting in sustained inhibition of cell migration, invasion, and adhesion. Through daily exposure to 0.1–0.4.1.4 T, 4.2 Hz fields for 6 h, a 31–46% extension in survival and a significant reduction was observed in lung metastases in MDA-MB-231 tumor-bearing mice was observed. Importantly, normal cells exhibited rapid recovery post-treatment cessation, highlighting the safety and potential clinical applicability of this non-invasive cytoskeletal-targeted method.

#### Dual and multiple-stimuli responsive systems

The development of single-response hydrogels has enabled targeted drug delivery under specific conditions. Yet these systems often struggle to account for the complex, synergistic effects of multiple variables within TME. Consequently, researchers have increasingly focused on designing dual- or multi-responsive hydrogel systems that can improve the accuracy and efficiency of drug delivery by integrating multiple physical and chemical stimuli-responsive mechanisms.

For instance, Xu et al. [89] developed a hydrogel system with photo/pH-response for the synergistic treatment of metastatic breast cancer and wound infections. Local sustained release of DOX and photothermal therapy can be achieved under acidic TME and NIR light irradiation. Meanwhile, this hydrogel system decomposed endogenous ROS into oxygen, alleviated hypoxia in the TME, and promoted wound healing. Fan et al. [90] developed a triple-responsive hydrogel (photo-/thermal-/enzyme) for synergistic therapy of postoperative tumor recurrence and wound infection. The nanoenzymes (including CuMnOx and CuO_2_) efficiently generated ROS within the TME and alleviated tumor hypoxia, thereby enhancing the efficacy of photodynamic therapy. The photothermal agent IR820 enabled low-temperature photothermal treatment under NIR irradiation, enabling the precise elimination of tumor cells without harming normal tissues. Furthermore, this hydrogel exhibited temperature-responsive and injectable properties, enabling sustained local drug release and minimizing systemic side effects. Additionally, its antibacterial properties help prevent postoperative infections, promote wound healing, and reduce the risk of tumor recurrence.

Hydrogel research for BC treatment has seen substantial advancements in recent years, including single-, dual-, and multi-response systems. These hydrogels could accurately release drugs under specified conditions by utilizing various response mechanisms, therefore offering multiple strategies for targeted BC therapy. Table 3 systematically summarizes stimulus-responsive hydrogels for drug delivery in BC and presents a comprehensive breakdown of drug release efficiencies across various response modes, serving as a reference for relevant research.

**Table 3:** Stimulus-responsive hydrogel for drug delivery in breast cancer

Response-strategy	Stimulus	Bioactive agents	Hydrogel	Releasing efficiency (time)	Ref
**Single-response**	pH	DOX	Chitosan-GA	pH = 5.8:81.33% (144 h) pH = 7.4:31.22% (144 h)	[91]
	Temperature	DOX	PLGA-PEG-PLGA	pH = 5.5:91.02% (240 h) pH = 7.4:80.19% (240 h)	[84]
	Redox	ICG/RRx-001	IR@CPgel	ICG/RR-001: ≥ 5 days	[92]
	Enzyme	PTX/CD44	PTX/Bio-NG	pH = 5.0:80.2% (48 h)	[93]
	Photo	DOX/aPD-L1	cSELP hydrogel	37 ℃: 8.3% (27 h) NIR-ON: 3.3% (3 min) NIR-OFF: 0.0% (3 min)	[94]
	Magnetic	Silk fibroin	Fe_3_O_4_ MNPs	Adriamycin:60% (4 h) Rifampicin: 70% (100 h)	[95]
	Biomolecule-glucose	BSA	Cationic chitosan	5 mM: 60%−80% 25 mM: 20%−40%	[96]
	Biomolecule-siRNA	LPR and Herceptin-HA	ALPR	pH = 5.5–80% (48 h) pH = 7.4–72% (48 h)	[97]
**Dual-response**	pH + Temperature	5-FU	Poly(N-Isopropylacrylamide)-carboxymethyl-chitosan	pH = 7.4 + 25 ℃: 65% (48 h) pH = 7.4 + 37 ℃: 55% (48 h) pH = 2.1 + 25 ℃: 75% (48 h) pH = 2.1 + 37 ℃: 65% (48 h)	[98]
	pH + Photo	DOX	Chitosan-β-glycerol phosphate disodium salt	pH = 4.5 + 4 w/cm^2^:90% (12 h) pH = 4.5 + 2 w/cm^2^:50% (12 h) pH = 4.5 + 0 w/cm^2^:30% (12 h) pH = 6.0 + 4 w/cm^2^:20% (12 h) pH = 6.5 + 4 w/cm^2^:15% (12 h) pH = 7.4 + 4 w/cm^2^:10% (12 h)	[99]
	pH + Redox	DOX	DSNGs	pH = 5 + GSH:90% (24 h) pH = 7.4-GSH 90% (120 h)	[24]
	pH + Magnetic	PTX + DOX	PEGylated Fe_3_O_4_ nanoparticles + α-Cyclodextrin	PTX: ACMF: 13% (5 min) DOX: ACMF: 63% (5 min)	[100]
	pH + Biomolecule	DOX	HA-BP-MOF	pH = 7.4:2.6% (97 h) pH = 6.0:5.7% (97 h) pH = 5.0:13.4% (97 h) pH = 7.4 + ATP:10.2% (97 h) pH = 5.0 + ATP:26.9% (97 h)	[101]
	Temperature + Photo	DOX	PNAm-PDAAu	pH = 5.0 + 37 ℃:40% (12 h) pH = 5.0 + 37 ℃ + NIR:81% (48 h) pH = 7.4 + 37 ℃:25% (12 h) pH = 7.4 + 37 ℃-NIR:40%(48 h)	[102]
	Temperature + Enzyme	DOX	4-vinylbenzyl chloride/trehalose	pH = 6.3:100% (4 h)	[103]
	Photo + Magnetic	DOX	Chitosan-poly-Fe_3_O_4_	pH = 7.4:50% (70 h)	[104]
	Enzyme + Magnetic	DOX	MMP2/MMP9-GelMA-Fe_3_O_4_	15%w/v:6% (48 h) 10%w/v:15% (48 h) 5% w/v:23.5% (48 h)	[105]
**Multiply-response**	Photo + Temperature + Enzyme	IR820	CMCI Gel	25 ℃: 21% (90 min) 45 ℃: 44% (90 min)	[90]

### Routes of drug administration

**Fig. 5:**
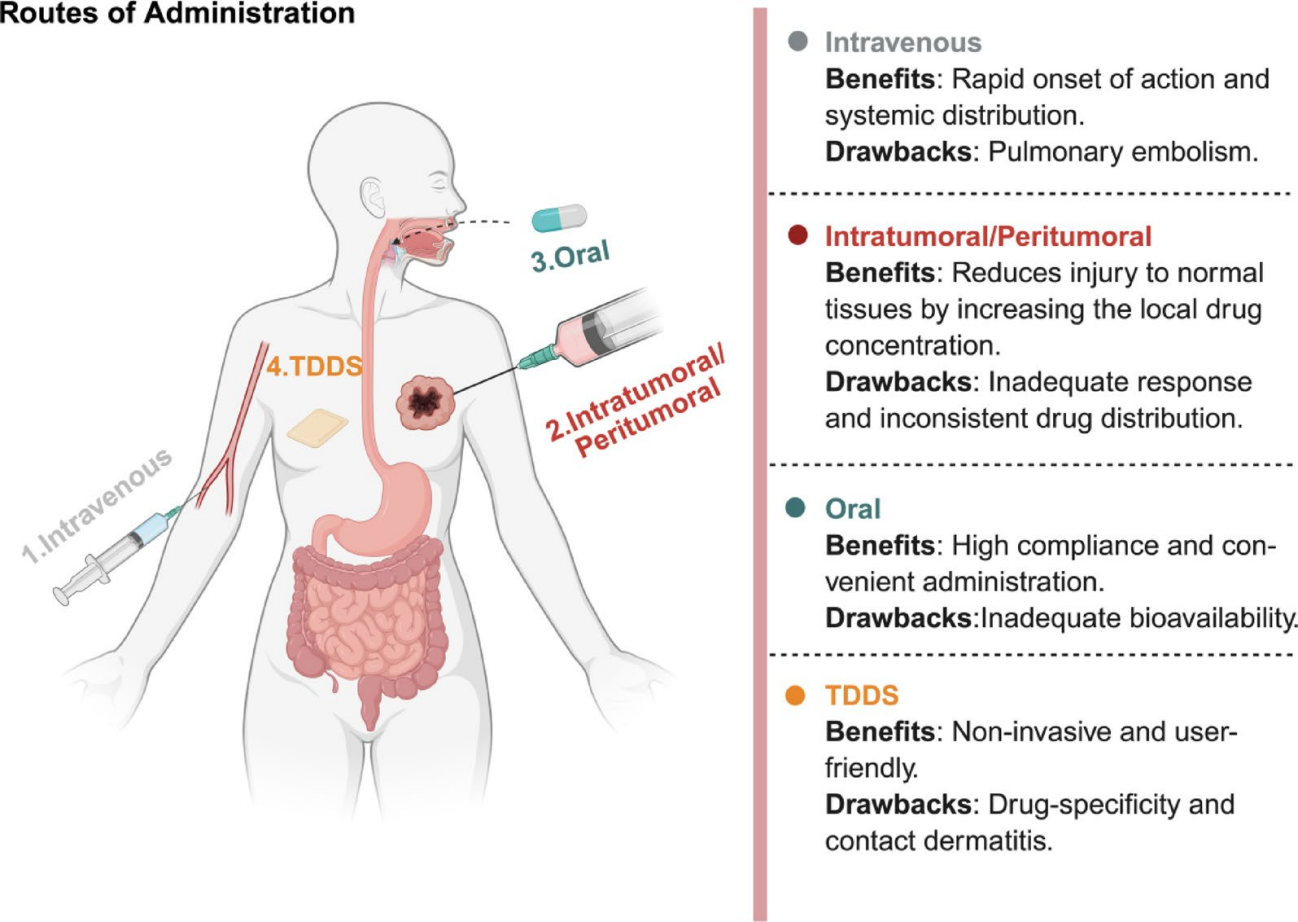
Benefits and drawbacks of the four primary administration routes of hydrogel

Drug delivery systems (DDS) aim to enhance therapeutic efficacy at target sites while minimizing off-target toxicity [106]. To achieve this goal, pharmacokinetic properties, tissue targeting, and molecular stability should be considered simultaneously. With the widespread use of biological agents such as nucleic acids, antibodies, and targeted small-molecule therapies, the challenges have become more complex and prominent. Due to adjustable physicochemical properties and adaptive interactions with biological interfaces, hydrogels are becoming a pivotal platform in drug delivery [107, 108]. Hydrogels have emerged as versatile platforms for systemic, local, and oral drug delivery, addressing the inherent limitations of each administration route. This section outlined the main drug delivery approaches on hydrogels. The primary administration routes explored include intravenous, intratumoral, and peritumoral injections, oral administration, and transdermal delivery (Fig. 5). The strategic selection of these routes aims to enhance targeting of therapeutic agents, minimize drug degradation, overcome physical barriers, prevent systemic side effects, and achieve sustained release, thereby improving the therapy efficacy.

#### Injectable hydrogel systems

Currently, drug delivery research and development based on hydrogel predominantly focus on local injection, particularly intratumoral and peritumoral injection. These systems utilize temperature-sensitive, ionic, or pH-responsive materials. Upon injection via syringe, the hydrogel rapidly undergoes in situ gelation in vivo, forming a "drug micro-library" that can persist for days to weeks. This strategy circumvents high tumor interstitial pressure, resulting in elevated local drug concentrations, reduced systemic toxicity, and the simultaneous delivery of chemotherapeutic agents, immune agonists, cytokines, or CAR-T cells. Consequently, it reshapes the tumor immune microenvironment, activates CD8^+^ T cells, and elicits abscopal effects. Efficacy has been demonstrated across various solid tumor models such as BC, colon cancer, and melanoma [109]. However, the clinical translation of intravenous hydrogels has been challenging. Macroscopic hydrogel particles pose a risk of embolism, while nanogel formulations face issues such as rapid clearance by the reticuloendothelial system, suboptimal tumor targeting, and uncontrolled in vivo gelation. These limitations have constrained the widespread adoption of intravenous hydrogel-based therapies [110]. As of now, no viable animal or clinical protocols have been established. Thus, future clinical translation of hydrogels will continue to prioritize local injections, while progress in intravenous delivery hinges on innovations in ultra-small nanocarriers, targeted modifications, and controlled-release mechanisms.

**Fig. 6:**
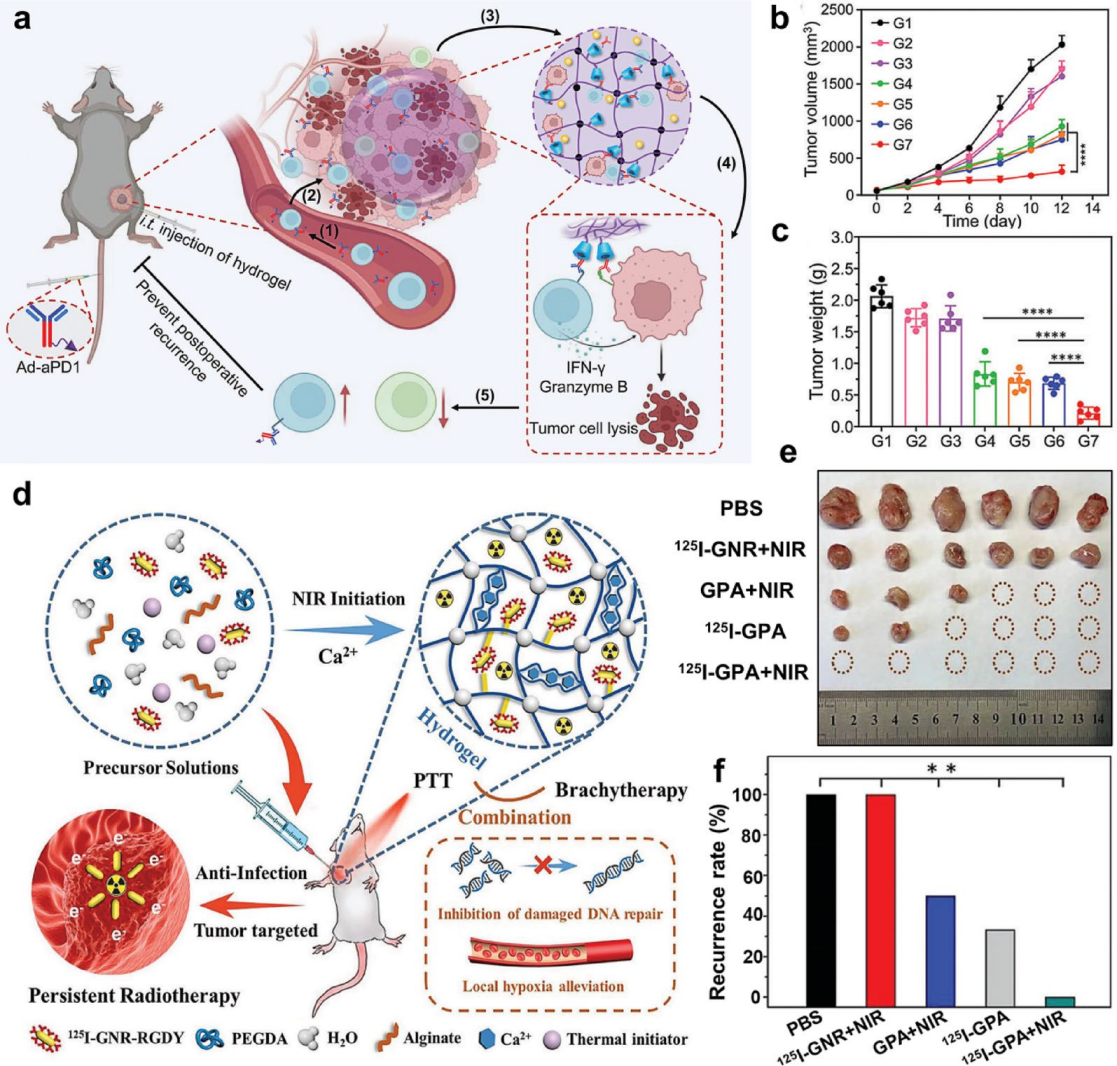
Schematic diagram of RER-T system enhancing T cell-mediated immunotherapy (**a**); The changes of tumor volume (**b**) and tumor weight **(c**) of mice under various treatments. (Adapted with permission from [111]. Copyright^©^2023 Wiley–VCH GmbH). Schematic diagram of the application of nanocomposite double-network GPA hydrogel to inhibit postoperative BC recurrence and wound infection by synergistic brachytherapy and PTT (**d**); The digital images of tumor (**e**) and recurrence rate (**f**) after various treatments. (Adapted with permission from [112]. Copyright^©^2022 Wiley–VCH GmbH)

Intratumoral injection enables the direct administration of hydrogels into tumor tissue, facilitating precise drug delivery. This approach is particularly suitable for treating multiple solid tumors, as it effectively stimulates a local immune response and enhances therapeutic outcomes. For example, Zhu et al. [111] developed an injectable β-cyclodextrin-alginate (ALG-βCD) supramolecular hydrogel to enhance T cell-mediated tumor immunotherapy by promoting the recruitment, engagement, and reinvigoration of effector T cells (Fig. 6a). This β-cyclodextrin-modified hydrogel exhibited excellent injectability and gelling ability. Its three-dimensional porous structure (pore size 100–200 μm) provided support for cell infiltration and sustained drug release. In the B16-F10 tumor model, a single intratumoral injection of this gel (containing CCL25 and Ad-aPDL1) can form an "immunomagnet" in situ, significantly increasing the proportion of CD8⁺ T cell infiltration in the tumor to 61% and triplicating the proportion of CCR9⁺CD8⁺ T cell subsets. The treatment ultimately achieved a tumor growth inhibition rate of 87.7%. (Fig. 6b and 6c). Complementarily, Wu et al. [112] reported a double-mesh hydrogel for intratumoral injection, effectively preventing tumor recurrence and wound infection by combining PTT and brachytherapy (Fig. 6d). SEM analysis confirmed that the hydrogel possesses a distinctive double-layer network structure characterized by interwoven nanofibers. In the 4T1 recurrence model, a single administration of ^125^I-GPA (100 µCi) reduced mean tumor volume to undetectable levels and achieved 0% recurrence (0/6 mice) at day 28, whereas PBS controls exhibited 100% recurrence (Fig. 6e). Tumor weight was virtually zero in the synergistic photothermal-brachytherapy group, demonstrating complete local tumor eradication. (Fig. 6f).

In contrast, peritumoral injection involves administering the hydrogel into the tissues surrounding the tumor, facilitating the gradual release of the drug and establishing a concentration gradient. This approach can attract and facilitate the migration of immune cells to the tumor site, thereby enhancing the efficacy of immunotherapy. For example, Liu et al. [113] developed a shear-responsive injectable supramolecular hydrogel system that sustainedly released pH-sensitive DOX-loaded GC-PF127 micelles for local tumor chemotherapy. The hydrogel, formed via inclusion complexation between cyclodextrin and micelles, exhibited injectability, rapid recovery, and controlled release of DOX/micelles. After peritumoral injection in H22 tumor-bearing mice, the hydrogel significantly enhanced DOX accumulation in tumor tissue while minimizing its distribution in normal organs, especially the heart. Compared to free DOX and intravenously injected micelles, a single hydrogel injection achieved superior antitumor efficacy, prolonged survival, and reduced systemic toxicity, including cardiotoxicity.

#### Oral delivery systems

Hydrogels are renowned for their exceptional physicochemical properties and biocompatibility. Their hydrophilic 3D network structure enables substantial water absorption and facilitates controlled drug release. This feature shields pharmaceuticals from the harsh gastrointestinal environment, enabling targeted delivery in response to physiological stimuli. Wang et al. [114] developed a pH-responsive PEG-PCL micelle-based hydrogel (DTX-micelle-hydrogel) for oral docetaxel delivery in BC therapy. This hydrogel facilitated targeted intestinal release by incorporating drug-loaded micelles (approximately 20 nm in size, with a drug loading of 7.76%) into a pH-sensitive 3D network. In vivo pharmacodynamic studies demonstrated that oral administration of the DTX-micelle-hydrogel (30 mg/kg) significantly suppressed tumor growth in a 4T1 breast cancer mouse model in comparison with the intravenous Taxotere^®^ group (p > 0.05) and the oral micelle group alone. Han et al. [115] further expanded the construction strategy for oral pH-responsive hydrogels and proposed a new scheme for a "polypill" based on semisolid extrusion 3D printing technology. This approach enabled precise drug release in the stomach (pH 1.2) or the intestine (pH 7.4) by adjusting the mass ratio of sodium alginate (SA) to carboxymethyl chitosan (CMCS). A SA:CMCS ratio of 1:3 facilitated drug release in the stomach, while a ratio of 3:1 targeted the intestine. The hydrogel system provided a more flexible and precise solution for personalized oral drug delivery, significantly improving the targeting and therapeutic effect of multi-drug combination.

#### Transdermal drug delivery systems (TDDS)

TDDS offer a non-invasive, controlled method for drug delivery, presenting distinct benefits in BC treatment by integrating post-radiotherapy skin repair with anticancer therapy. TDDS efficiently deliver anticancer drugs directly to the tumor site, allowing for controlled release and precise local drug regulation. which can improve therapeutic efficacy and minimize systemic side effects. Ravi et al. [116] developed a β-cyclodextrin-hyaluronic acid composite nanogel to address the systemic toxicity challenge posed by traditional chemotherapy by pH-responsive controlled release of DOX. The transdermal permeability and pH 5.5-targeted release enabled precise epidermal drug delivery. Building on this, Zhang et al. [117] extended the applications of hydrogels to postoperative scenarios by designing an implantable silk fibroin/perfluorocarbon (SF/PF) hydrogel. The system improved radio-sensitization by maintaining oxygen levels and releasing DOX. It combined an antioxidant bioadhesive patch with gold nanorods and gallic acid to reduce radiation-induced dermatitis, creating a comprehensive approach for tumor treatment and skin repair. Meanwhile, the team introduced a dual-functional hydrogel composed of lipoic acid, arginine, and silk fibroin (LA/Arg/SF), addressing the constraints of single-scenario applications [118]. This injectable hydrogel filled tumor resection cavities, suppressing recurrence through chemotherapy-radiation synergy and ROS scavenging. Concurrently, the adhesive patch facilitated angiogenesis via arginine release, expediting the healing of radiation-impaired wounds and establishing a "tumor suppression-tissue repair" dual closed loop. Repeated exposure to chemotherapeutic drugs promotes the expansion of tumor-initiating cells (T-ICs), significantly contributing to chemoresistance and cancer metastasis. Ji et al. [119] developed a TME-responsive hydrogel patch for targeted delivery of the LSD1 inhibitor GSK-LSD1 to modulate tumor-initiating cells (T-ICs) in TNBC. The engineered "epi-gel" promoted T-IC differentiation and reversed chemoresistance to multiple agents (e.g., 5-FU, paclitaxel); it also triggered an IFN-β-mediated immune response, thereby collectively inhibiting tumor progression, recurrence, and metastasis with minimal systemic toxicity.

### Combination therapies

Traditional monotherapy, whether chemotherapy or targeted therapy, faces limitations due to multidrug resistance caused by tumor heterogeneity and off-target toxicity [120, 121]. Additionally, the effectiveness of monotherapy tends to diminish over prolonged treatment cycles. Therefore, hydrogel-based synergistic therapeutic approaches, involving the controlled co-delivery of multiple therapeutic agents in a spatiotemporal manner, are increasingly recognized as a pivotal strategy to overcome adaptive drug resistance in tumors.

**Fig. 7:**
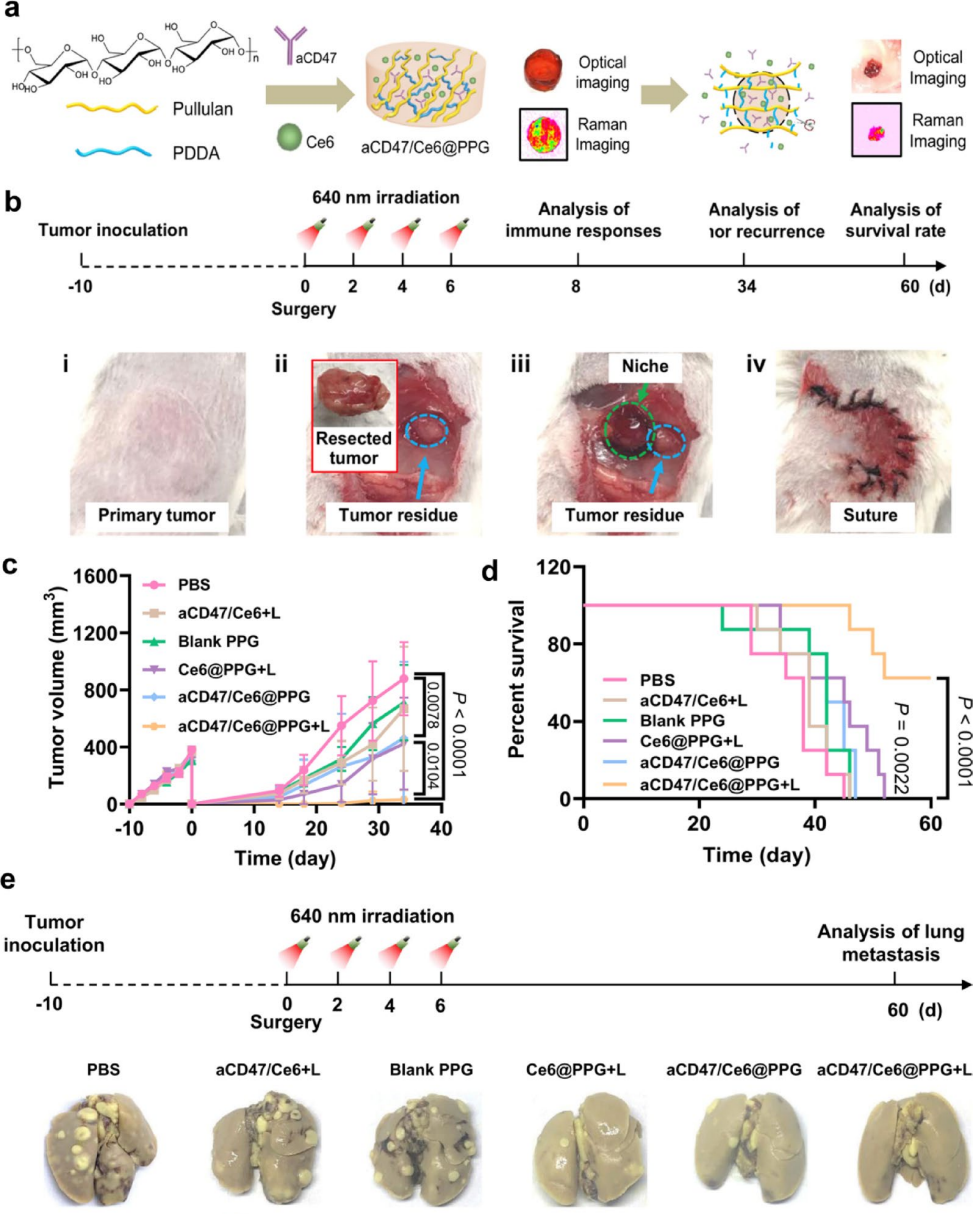
Schematic illustration of aCD47/Ce6@PPG hydrogel for the sustained co-delivery of photosensitizers and ICB antibodies (**a**); the process of the surgery, including (i) tumor volume reached ~300 mm^3^, (ii) tumor resection, (iii) implantation of the hydrogel, and (iv) suture (**b**); the changes of tumor volume (**c**) and survival percentages of the mice under various treatments (**d**); schematic diagram of experimental design of aCD47/Ce6@PPG hydrogel for prevention of lung metastasis and photos of representative lung tissues in different groups (**e**). (Adapted with permission from [122]. Copyright^©^2022, Springer Nature)

Hydrogel-based synergistic therapy leverages the unique properties of hydrogels to combine multiple therapeutic modalities and components, thereby eliciting enhanced therapeutic outcomes compared to individual treatments. Hydrogels can serve as versatile drug delivery vehicles, encapsulating a variety of pharmacological agents and facilitating their controlled, on-demand release in response to specific environmental cues, such as changes in temperature, pH, or light exposure. The concurrent delivery of distinct therapeutic agents can yield synergistic pharmacological effects. Furthermore, hydrogels can be integrated with physical therapies, including PDT, PTT, and sonodynamic therapy (SDT), to achieve combinatorial treatment approaches. The photothermal conversion material in the hydrogel generates heat upon NIR light irradiation, killing tumor cells. Combining ICB therapy with PDT holds great potential in treating immunologically “cold” tumors. For example, Zhang et al. [122] developed a ROS-responsive and Raman-traceable hydrogel (aCD47/Ce6@PPG) that co-loads the photosensitizer Ce6 and the immune-checkpoint antibody anti-CD47 (Fig. 7a). Raman images and an in vivo imaging system verified that PDDA-mediated ROS depletion can regulate hydrogel degradation and achieve controllable, long-lasting drug release. The 4T1-luc incomplete excision mouse model (Fig. 7b) demonstrated that this treatment protocol remodeled the tumor immune microenvironment. A single administration of the aCD47/Ce6@PPG hydrogel combined with irradiation nearly completely prevented tumor recurrence within 34 days, with a recurrence rate of only 28.6%. Importantly, this combination therapy (aCD47/Ce6@PPG + L) significantly inhibited tumor growth (Fig. 7c) and effectively improved the mouse survival rate (Fig. 7d) compared to the control group. Notably, this combination therapy also had a strong tumor-inhibitory effect in lung metastasis models (Fig. 7e).

**Fig. 8:**
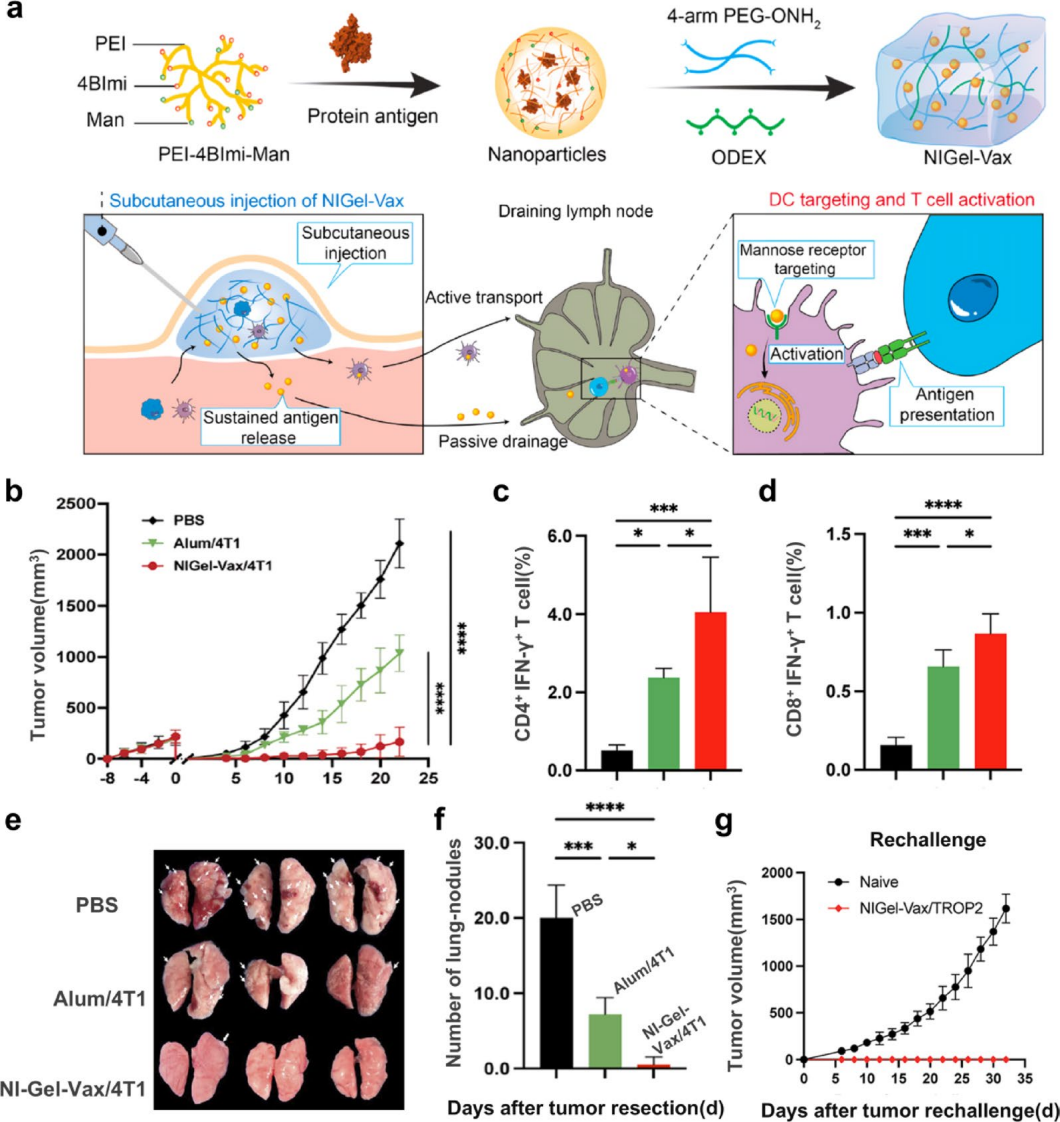
Schematic diagram of NIGel-Vax synthesis and anti-tumor immune mechanism (**a**); The changes of tumor volume after various treatments (**b**); The proportion of CD4^+^ IFN-γ^+^ (**c**) and CD8^+^ IFN-γ^+^ T (**d**) cells in the peripheral blood of the mouse after various treatments; The number of metastatic lung lesions visible to the naked eye in different groups (**e**) and statistical plot of visible metastases (**f**); The changes of tumor volume in rechallenged model (**g**). (Adapted with permission from [123]. Copyright^©^2024 American Chemical Society)

**Fig. 9:**
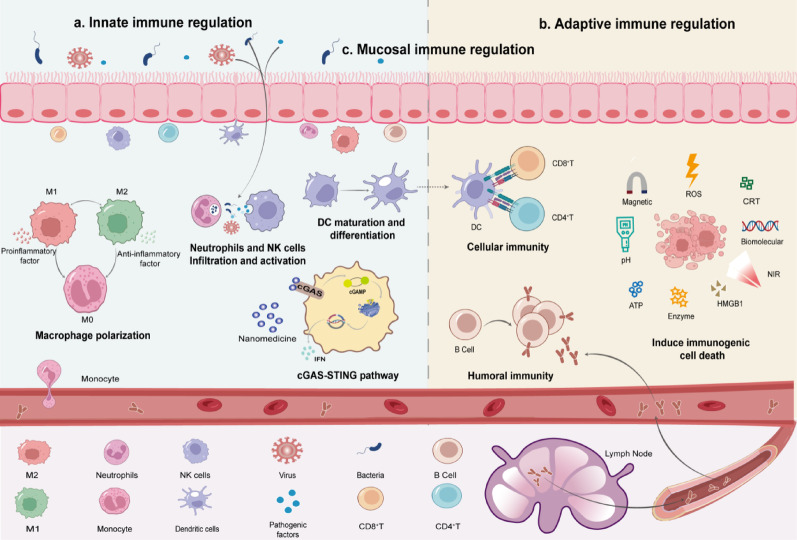
Hydrogel-mediated immunomodulatory mechanisms in BC therapy. This mechanistic map outlines the mechanisms by which hydrogel-based nanomaterials modulate the immune system and exert diverse immunotherapeutic effects across three aspects. **a**. Innate immune regulation: regulating macrophage polarization; recruiting and activating neutrophils; inducing DCs maturation and activation; regulating the cGAS-STING pathway. **b**. Adaptive immune regulation: inducing T-cell maturation and differentiation; inducing B-cell maturation and differentiation, and production of specific antibodies; inducing immune cell death, and releasing damage-associated molecular patterns. **c**. Mucosal immune regulation: releasing nano-drugs from hydrogel to strengthen the skin-mucosal barrier against cancer cells, viruses, bacteria, or pathogenic factors, etc

Breast-conserving surgery is a pivotal approach for early BC treatment. Reducing the toxic side effects of subsequent radiotherapy remains a pressing clinical challenge. Liu et al. [123] designed an injectable nanogel vaccine (NIGel-Vax) for the co-delivery of patient-derived tumor lysate antigens and a STING agonist adjuvant hydrogel for immunotherapy after breast-conserving surgery (Fig. 8a). This hydrogel exhibited tunable rheological properties, injectability, and self-healing capabilities. The three-dimensional porous structure, characterized by a pore size of 6–20 μm, along with an adjustable in vitro degradation time ranging from 7 to 27 days, facilitated the continuous and sustained release of nanoparticles. The 4T1 BC recurrence model demonstrated that a single subcutaneous injection of NIGel-Vax, using the patient's own tumor lytic protein as an antigen, achieved up to 92% tumor growth inhibition (Fig. 8b), significantly outperforming the traditional Alum adjuvant group. Mechanistically, the vaccine substantially expanded peripheral blood CD4^+^ IFN-γ^+^ and CD8^+^ IFN-γ^+^ effector T cell subsets by continuously releasing nano-antigens and targeting lymph nodes (Fig. 8c and 9d). Notably, the vaccine effectively suppressed distant metastasis after surgery, reducing the number of macroscopic pulmonary metastatic nodules to nearly zero (Fig. 8e and 9f). Importantly, with tumor rechallenge, mice that cleared tumors resisted primary tumor rechallenge (Fig. 8g), indicating that the nano-gel platform not only inhibited residual lesions after surgery but also induced long-lasting systemic immune memory. These findings suggested that NIGel-Vax could provide a simple and powerful translational immunotherapy regimen for patients at high risk of recurrence after surgery, such as those with TNBC.

The design of hydrogels represents a multifaceted and pivotal area of research. Effectively incorporating diverse pharmacological agents or engineering the structural properties of hydrogels necessitates addressing a myriad of complex technical obstacles. To consolidate and synthesize the current state of knowledge, we have systematically reviewed the various drug-loading strategies, response mechanisms, and administration modalities of hydrogels, and compiled representative examples in Table 4.

**Table 4:** Hydrogel-based nanomaterials combined various design strategies for breast cancer treatment

Year	Drug	Stimulus	Hydrogels	Drug-administration	Cell-lines	Disease type	Ref
2024	PTX	Temperature	PTX@HSA-HA NPs-(PTX NPs@Gel)	Intratumoral	MCF-7; 4T1	Breast tumor	[124]
2022	DOX	Temperature + Redox	Gel-^shKIAA^RPDNs-DOX	Intratumoral	4T1	Breast tumor; lung metastases	[74]
2020	DOX	pH + Redox	DSNGs-RGD-DOX	Intratumoral	MDA-MB-231	Breast tumor	[24]
2019	aCD47	Photo	aCD47/Ce6@PPG	Intratumoral	4T1	Breast tumor; lung metastases	[122]
2022	DOC	Photo	Poly (ethylene glycol) (PEG)-DTXL-μCGP	Intratumoral	MDA-MB-231	Advanced BC; lung metastasis	[38]
2024	OVA/TROP-2	None	PEI-4BImi-Man-NIGel-Vax	Intratumoral	4T1	Breast tumor; lung metastases	[123]
2022	Cyclopamine + aCD47	None	thiolated chitosan (CS-SH)-aCD47/C@Gel	Intratumoral/Peritumoral	4T1	Breast tumor; lung metastases	[125]
2019	Trastuzumab	None	γ-PGA-MA/4-arm PEG-SH-Her/Zn-hydrogel	Peritumoral	BT-474	Breast tumor	[40]
2023	aPD-1	Temperature	TCCaGM hydrogel	Intratumoral	4T1	Breast tumor; lung metastases	[126]
2025	Abe + NLG919	pH	Abe-NF(gel)	Intratumoral	4T1	Breast tumor; lung metastases	[27]
2024	ELE	None	ELE@LPNPs@Gel	Intratumoral	4T1; MDA-MB-231	Postoperative cancer recurrence; Lung metastases	[30]
2021	iLSD1 + 5-FU	Redox + Biomolecule	PVA/TSPBA-iLSD1 + 5-FU@Gel	Intratumoral	4T1	Multidrug-Resistant TNBC	[119]
2022	SAHA +3MA	pH	Gel-OS/SAHA@3MA@MBGN hy-drogel (GOSAM)	Intratumoral	MDA-MB-231	Post-surgical relapse of TNBC	[127]
2024	5-FU	pH	[Cho][Ala]/[Cho][Pro]-based ionic hydrogel	TDDS	MCF-7	BC local recurrence	[60]
2021	IR820 + MPs	Temperature + Photo	IR820/Mgel + L@Gel	Intratumoral	4T1	Post-surgical tumor recurrence; breast reconstruction	[128]
2023	DOC + CD47siRNA	Redox + Biomolecule	DTX/USIO@PEI NGs/siCD47@ CM	Intratumoral	4T1	Breast tumor; lung metastases	[129]
2024	Resiquimod + Cabazitaxel	Photo + ROS	PVA/TSPBA-iGEL	Intravenous/Peritumoral	4T1; 4T1-luc	Breast tumor	[130]
2014	DOC	pH	DTX-micelleehydrogel	Oral	4T1	Breast tumor	[114]
2024	Esketamin + Cisplatin	Temperature	PDEH@Gel	Intratumoral	4T1	Painless tumor immunotherapy	[129]
2021	Gemcitabine + D-1-methyltryptophan	pH + Biomolecule	GEM/D-1MT gels	Intratumoral	4T1	Breast tumor; lung metastases	[46]
2023	Decitabine + gambogic acid	Photo	POLY-PEG-DMA/SER-MA hydrogels	Intratumoral	4T1; 4T1-luc	Postsurgical wound management; prevention of TNBC recurrence	[131]
2025	CDs/CA170/LPS	Biomolecule	TTF-L-C@Gel	Intratumoral	4T1	Breast tumor; local recurrence	[132]

## Immunoregulation of hydrogel-based nanotherapeutics

The immune system plays a vital role in protecting the body from pathogens and cancer cells. Hydrogels, serving as smart carriers for immune modulation, function by finely tuning various immune signaling pathways. Here, we analyze in detail the immunomodulatory approach through which hydrogels collaboratively control the TME. The immunomodulatory approach mainly focuses on three aspects: innate immune regulation, adaptive immune activation, and mucosal immune enhancement (Fig. 9).

### Regulation of innate immune signaling pathways

The innate immune system, as the body's first line of defense, plays a dual role in tumor immune surveillance by detecting pathogen-associated molecular patterns (PAMPs) and damage-associated molecular patterns (DAMPs) via pattern recognition receptors (PRRs). During tumorigenesis, it swiftly identifies abnormal molecular features from tumor cells, such as exposed calreticulin and high mobility group protein 1, and activates signaling pathways. This activation occurs through PRR family members, including Toll-like receptors (TLRs) and NOD-like receptors (NLRs), which engage both MyD88-dependent and independent pathways. These pathways, in turn, activate NF-κB and interferon regulatory factors. The activation of innate immune pathways within TME can elicit a multifaceted response. This cascade promotes the infiltration of innate immune cells, such as macrophages and natural killer cells, into the TME, directly eliminating malignant cells through antibody-dependent cytotoxicity and phagocytosis. Concurrently, the induction of an inflammatory cytokine storm and complement cascade creates a critical time window for antigen-presenting cells (APCs) to present tumor-specific antigens to T cells, thereby bridging adaptive immune responses. Hydrogel-based DDS can enhance the tumor recognition and clearance efficiency of the innate immune system by regulating the spatiotemporal activation of these signaling pathways. For instance, the sustained release of Toll-like receptor (TLR) agonists can maintain the continuous activation of DCs, while MMP-responsive hydrogels can achieve precise local release of IL-1β.

The modulation of macrophage polarization, through the regulation of signaling pathways involving factors such as TNF and PGE2, can influence the expression of inflammation-related molecules, including CCL2, CCL5, and PD-L1. This, in turn, can impact TME, as M2-polarized macrophages have been shown to accelerate tumor progression by suppressing T cell activation and promoting angiogenesis. Gong et al. [133] developed a multifunctional injectable hydrogel (OC@ε-PL-SATO) with fast gelation (< 30 s) and continuous hydrogen sulfide (H_2_S) release, which effectively induced M0 to M1 macrophage polarization and enhanced the inflammatory response. Transcriptome analysis reveals that the release of H_2_S from the OC@ε-PL-SATO hydrogel inhibited the LPS-mediated inflammatory response in RAW264.7 cells by disrupting the PI3K/Akt signaling pathway and NF-κB activation. Chen et al. [125] developed an orthotopic hydrogel loaded with an anti-CD47 antibody (aCD47) to boost M1-like macrophage activity and suppress M2-like macrophages by continuously releasing aCD47, thereby inducing an anticancer immune response. Tang et al. [134] constructed a Gd-metallofullerol-based nanodrug delivery system that synergistically enhanced chemotherapy efficacy by inducing macrophage polarization toward the M1 phenotype. Gd-metallofullerenol stimulated a Th1 immune response by promoting M1 macrophage polarization, while DOX directly killed tumor cells through its cytotoxic effects. This synergistic effect greatly enhanced the anti-tumor immune response. Zhang et al. [126] developed an advanced hydrogel (TCCaGM) that mitigated hypoxia and acidic TME, increased catalase levels in tumor cells, and reduced CD47 expression, thereby recruiting and activating intratumoral DCs. This process enhanced macrophage phagocytosis and initiated tumor-specific CD8^+^ T cell responses.

DCs are crucial antigen-presenting cells that regulate immune tolerance and autoimmune responses by presenting antigens to lymphocytes. Hydrogel-based antitumor drugs significantly modulate immune regulation by altering DC maturation, activation, and antigen presentation, thereby enhancing DC-mediated immune responses. Ke et al. [135] constructed a DCs activation hydrogel based on bifunctional fusion membrane nanoparticles (FM-NPs), which was composed of autologous tumor cell membranes and mycobacterium phlei membrane extracts. These FM-NPs interacted with chemotactic DCs within the hydrogel, promoting their maturation. In the 4T1 mouse model, this hydrogel facilitated DCs maturation in tumor-draining lymph nodes and induced the migration of effector memory T cells to TME. Xu et al.[136] developed a bioink DC vaccine using 3D printing technology to encapsulate DCs and tumor cellular vesicles that overexpressed PD1-CVs for cancer immunotherapy. The PD1-CVs promoted DC maturation and inhibited PD-L1 expression in mature DCs and tumor cells, thereby synergistically enhancing DC-mediated antitumor immune responses. In breast tumor models, this personalized 3D DC vaccine, designed for precise administration following tumor resection, significantly reduces tumor recurrence and prolongs survival.

Neutrophils play a crucial role in the innate immune system [137]. Mao et al. [138] embedded Ag/Ag@AgCl/ZnO hybrid nanostructures into hydrogels using a simple two-step method, and then incorporated ZnO nanostructures via NaOH precipitation. In vivo experiments showed that the release of Ag^+^ and Zn^2+^ could stimulate the immune response, increase the number of leukocytes and neutrophils (2 ~ 4 times that of the control group), thereby producing synergistic antibacterial effects and accelerating wound healing. Zhu et al. [139] engineered an intraoperative cavity hydrogel that exploits neutrophil-extracellular-trap (NET) formation to achieve NET-specific, long-term release of anti-TGF-β after breast-cancer resection. In vivo, this selective suppression of TGF-β blocked epithelial-mesenchymal transition, reversed NET-driven immunosuppression, and markedly reduced post-surgical recurrence while displaying excellent biocompatibility.

The cGAS-STING pathway is a critical component of the innate immune system, with profound implications for tumor biology, epidemic immunity, and autoimmune disorders [140]. Lv et al. [141] reported that manganese (Mn^2^⁺) was a potent activator of the cGAS-STING pathway, which is essential for innate and adaptive immune responses against tumors. Mn^2^⁺ promoted DCs and macrophage maturation, enhanced CD8⁺ T cell and NK cell activation, and increased memory T cell formation in a cGAS-STING-dependent manner. In mouse models, Mn^2^⁺ treatment significantly inhibited tumor growth and metastasis, and synergistically enhanced the efficacy of anti-PD-1 immunotherapy even at reduced antibody doses. Furthermore, a phase 1 clinical trial showed that Mn^2^⁺ combined with anti-PD-1 and chemotherapy revived immunotherapy responses in patients with advanced metastatic solid tumors, with manageable safety and induction of type I interferons. Tian et al.[142] developed a self-oxygenated hydrogel drug delivery platform, ALG@TPN, which triggered dual DNA damage to activate the cGAS-STING pathway and inhibit cancer-associated fibroblasts (CAFs). This approach enhanced immune cell responses and infiltration within the TME, induced robust anticancer immunity, and eliminated both the cellular and extracellular components of the tumor niche, demonstrating significant promise for cancer therapy.

### Modulation of adaptive immune responses

Adaptive immunity is characterized by high specificity and immune memory, involving humoral immunity mediated by B lymphocytes and cellular immunity mediated by T lymphocytes. This system can regulate lymphocyte function, further enhance B cell antibody secretion, and promote T cell activation and tumor infiltration. Simultaneously, it induces the release of tumor-associated antigens and enhances the antigen presentation efficiency of DCs, thereby activating specific immune responses. This dual mechanism offers new avenues for improved tumor immunotherapy strategies and molecular targets.

Hydrogel-based drugs modulate lymphocyte immune responses, which are crucial for both cellular and humoral immunity in tumor immunotherapy. Yin et al. [73] developed an immune-enhancing injectable hydrogel loaded with esketamine and DDP, which can promote painless immunochemotherapy to inhibit breast cancer growth. This research found that esketamine, combined with DDP co-loaded into a poloxamer hydrogel, induced local immunity by increasing mature DCs and activated T cells, demonstrating a powerful anti-tumor effect. Zheng et al. [143] developed an acid-sensitive hydrogel (PEG-CaCO_3_@CUR) that disrupts endoplasmic reticulum calcium recovery by inhibiting the SERCA pump and activating the IP3R channel, thereby elevating cytoplasmic Ca^2^⁺ levels to over 5 μM. The excess Ca^2^⁺ entered mitochondria via the MCU channel, causing oxidative phosphorylation uncoupling and a ROS surge (8.2 times higher than the control), ultimately triggering a robust ICD effect through a calpain-ROS positive feedback loop. This approach enhanced intratumoral CD103^+^ DC maturity by 73.5%, increased CD8^+^ T cell infiltration by 4.2-fold, and synergized with PD-1 inhibitors to enhance anticancer effects.

### Induction of mucosal immune responses

The mucosal immune system is the host's primary defense against pathogen infiltration. Drug delivery systems, including nasal, transdermal, and oral, play a crucial role in modulating immune responses by activating the epithelial barrier. Notably, transdermal hydrogels enhance wound healing by promoting M2 macrophage polarization through matrix programmability and activating DCs to mediate tumor immune surveillance via the TLR4/MyD88 pathway.

Based on the targeted delivery advantages of transdermal hydrogels, Song et al. [119] developed epigenetic-immune synergistic hydrogel patches for chemotherapy-resistant TNBC. The system used a thioketone-crosslinked hyaluronic acid scaffold to synthesize a ROS-responsive matrix (critical gel concentration of 0.8%), precisely loading the LSD1 inhibitor (iadademstat) and 5-FU for dual-controlled release. In animal studies, a single application reduced the volume of drug-resistant TNBC xenografts by 82%, increased CD8^+^ T cell infiltration density by 4.5 times via the CCL5/CXCL10 axis, and achieved a 67% complete remission rate post-PD-1 blockade, outperforming traditional intravenous methods. This approach surpassed the unidirectional action of conventional transdermal preparations, enabling the 3D regulation of epigenetic reprogramming, chemosensitization, and immune activation, and offered a viable engineering solution to reverse drug resistance in solid tumors.

### Mechanisms of TME modulation by hydrogel-based systems

**Fig. 10:**
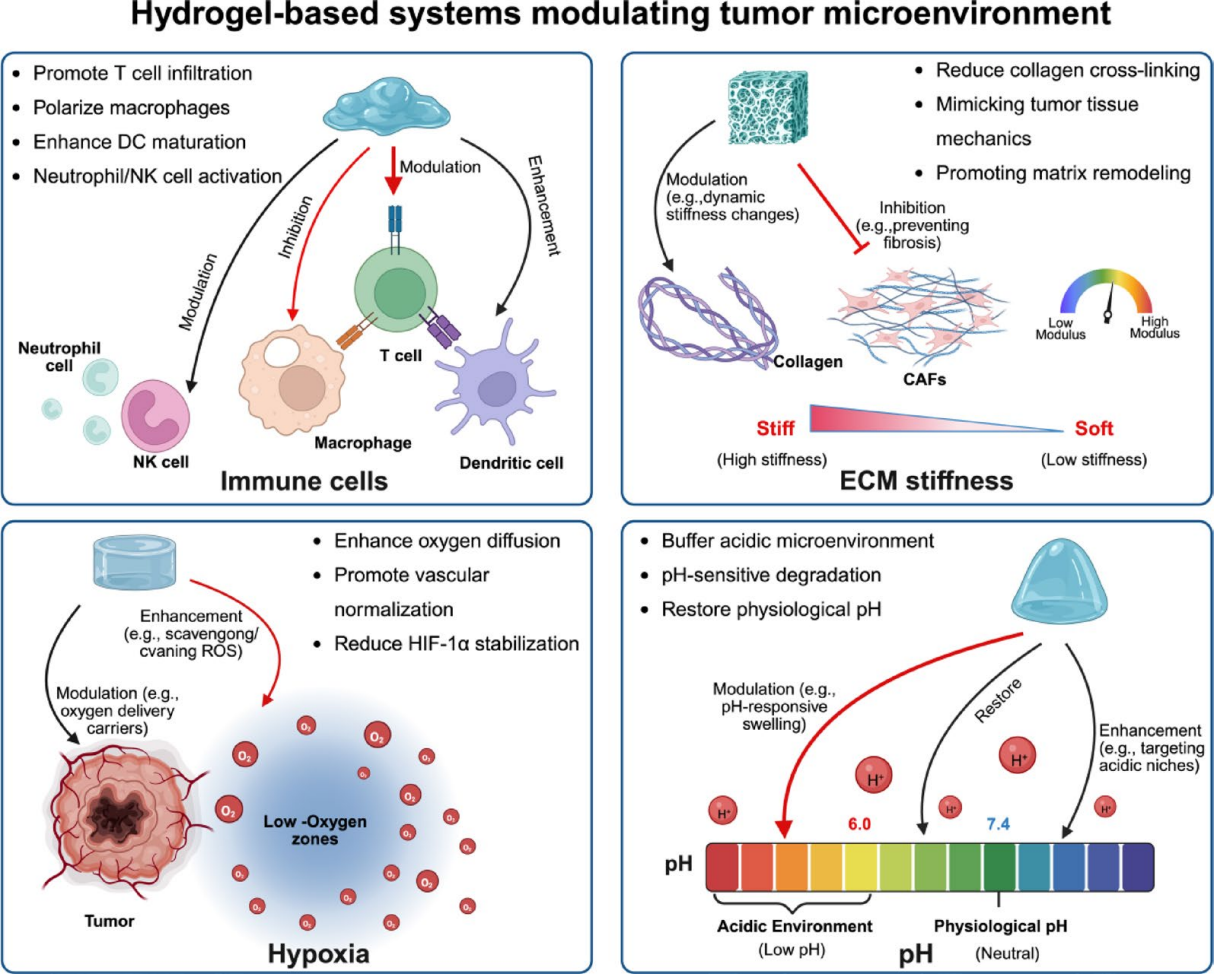
Schematic illustration of the multifaceted remodeling of TME by an intelligent hydrogel-based nanomaterials

The complex and immunosuppressive TME is characterized by dysfunctional immune cell populations, a dense ECM, hypoxia, and acidosis, all of which constitute a major barrier to breast cancer therapy [144]. Nanocomposite platforms based on hydrogels, a powerful local drug-delivery system, can achieve multifaceted reshaping of the TME. As shown in Fig. 10, the modulation mechanisms can be categorized into four aspects:

(1) Remodel the tumor immune microenvironment: The localized and sustained release of immunomodulatory drugs from the hydrogels directly targets the cellular components of the TME. For instance, the hydrogels loaded with agents can repolarize immunosuppressive M2-type tumor-associated macrophages (TAMs) towards the tumoricidal M1-type. Simultaneously, the released compounds promote the infiltration, activation, and proliferation of cytotoxic T lymphocytes (CTLs), thereby converting an immunologically "cold" TME into a "hot" one conducive to antitumor immunity.

(2) ECM remodeling: The dense collagen-rich ECM and high interstitial fluid pressure (IFP) in tumors significantly hinder drug diffusion. An enzyme-responsive hydrogel platform can be engineered to release matrix-degrading enzymes (e.g., collagenase) upon injection. This localized enzymatic activity specifically degrades stromal components, reduces tissue stiffness, and lowers IFP, thereby breaking down the physical barrier and significantly improving the penetration and uniform distribution of co-delivered therapeutic agents deep into the tumor parenchyma.

(3) Hypoxia alleviation: Tumor hypoxia, a consequence of abnormal vasculature, drives treatment resistance and immune suppression. Hydrogel-based nanomaterials can load oxygen-generating materials (e.g., calcium peroxide) or ROS-scavenging catalysts. By locally generating oxygen or clearing excessive ROS, the hydrogel alleviates hypoxia, inhibits the stabilization of hypoxia-inducible factor-1α (HIF-1α), and promotes vascular normalization. This amelioration of the aberrant metabolic environment enhances the efficacy of both chemotherapy and immunotherapy.

(4) Acidic pH-responsive drug release: The glycolytic metabolism of cancer cells creates an acidic extracellular pH, which suppresses immune cell function. Hydrogel-based systems can be designed with pH-sensitive chemical bonds (e.g., hydrazone bonds). In the acidic TME, these bonds cleave, leading to hydrogel swelling or dissolution. The released drugs can adjust the acidic pH, neutralize the immunosuppressive acidosis, and further enhance immune cell activity.

## Biomedical applications and clinical translation of hydrogels

Hydrogels offer versatile applications in theranostics of breast diseases. As innovative biomaterials, their injectability, dynamic mechanical adaptability, and responsiveness to microenvironments enable precise drug delivery and the seamless integration of diagnostics and therapy. In tissue engineering, GelMA-based 3D printable hydrogels can precisely fill irregular tissue defects and guide adipose stem cell differentiation by modulating matrix stiffness [145]. In tumor research, hydrogels derived from decellularized matrices facilitate the creation of 3D organoid co-culture systems with CAFs and TAMs, effectively modeling IL-6/STAT3-mediated chemoresistance in tumor niches. The following section will explore the clinical applications of hydrogels in BC from three dimensions.

### Wound healing and tissue regeneration

As a material with excellent biocompatibility, the clinical applications of the hydrogel are gradually expanding from traditional fields into tumor treatment. Early studies primarily focused on the care of chronic wounds, such as diabetic foot ulcers, by promoting healing through a moist environment and sustained-release antibacterial agents. In recent years, advances in materials science have enabled hydrogels to exhibit unique value in postoperative care for BC, thanks to their design flexibility and functional diversity.

For instance, Zhang et al. [118] developed a natural, injectable, and attachable dual-morphology PolyLA/Arg/SF hydrogel. PolyLA12SF4 gel with low LA content demonstrated shear-thinning and self-healing properties, effectively filling tumor resection cavities. This gel completely prevented postoperative recurrence by enhancing radiotherapy and continuously releasing LA/Arg in the 4T1 mouse model. Furthermore, the PolyLA12SF4 patch with high LA content, markedly accelerated the healing of radiation-induced skin injuries and infected wounds. This was attributed to its strong ROS scavenging, NO-enhanced vascularization, and broad-spectrum anti-drug-resistant bacteria (MRSA) efficacy. Chen et al. [89] developed a multifunctional hydrogel system for combined reoxygenation and chemo/photothermal therapy against metastatic breast cancer and wound infection. The hydrogel enabled controlled release of doxorubicin (DOX) and maintained prolonged drug concentration at the lesion site, thereby enhancing therapeutic efficacy and minimizing systemic toxicity. Concurrently, the photothermal capability of PBCEC synergized with DOX to enhance tumor eradication. Additionally, the hydrogel decomposed exogenous CaO₂ into O_2,_ mitigating tumor hypoxia and aiding in the healing of postoperative infected wounds. This facilitated a reoxygenation-chemotherapy-photothermal synergistic approach for treating metastatic breast cancer and wound infections. However, with the ongoing advancement of nanomaterial technology, research on breast cancer hydrogels has gradually moved beyond the traditional scope of "inhibiting tumor recurrence" and "promoting wound healing". Currently, the development of hydrogels with both anti-tumor activity and structural support functions to maintain postoperative breast shape is a new research hotspot and development direction in this field.

### Breast reconstruction

BC poses a significant global threat to women's health, with persistently high incidence and mortality rates. Surgical resection remains the primary treatment, yet the risks of local recurrence and breast tissue defects present urgent clinical challenges. In response, researchers have developed innovative hydrogel platforms to address postoperative tumor treatment and breast reconstruction simultaneously.

**Fig. 11:**
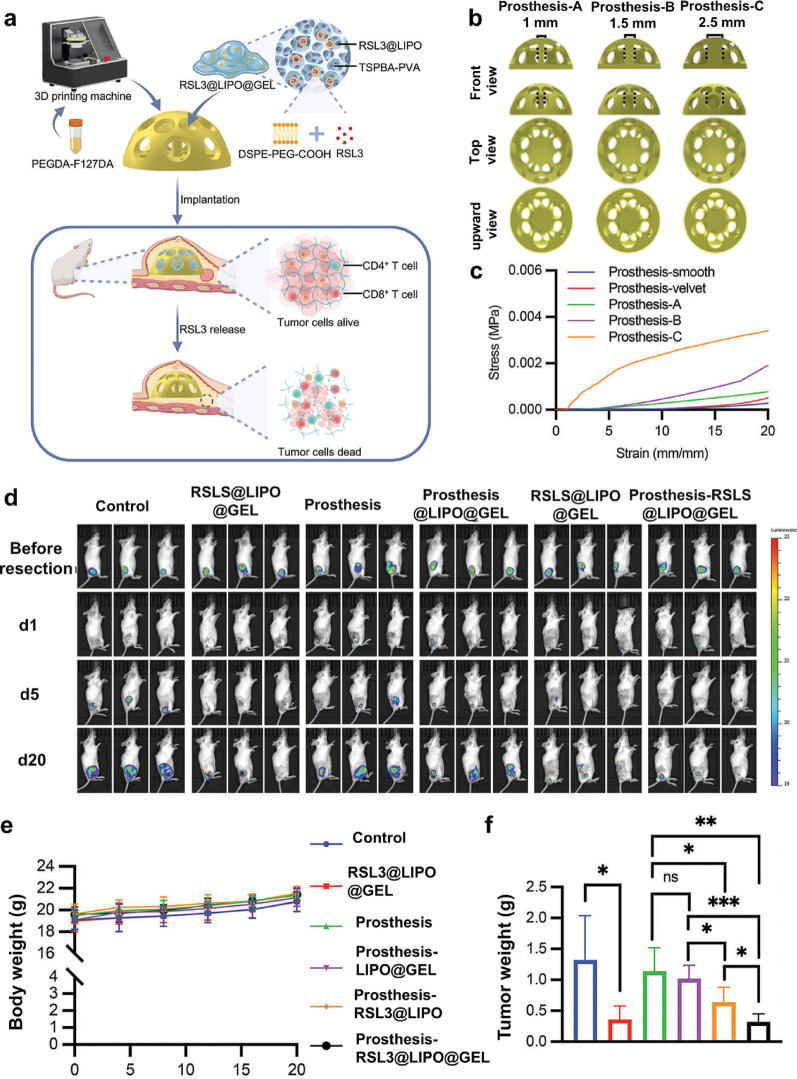
Schematic illustration showing the preparation of smart custom-made breast prosthesis and its application in breast reconstruction and treatment for recurrence (**a**); 3D-printed models of breast prostheses (**b**) and comparison of compressive modulus (**c**); In vivo bioluminescence images of 4T1 breast tumor-bearing mice at different time points (**d**); The changes of tumor weight (**e**) and body weight (**f**) after various treatments. (Adapted with permission from [147]. Copyright^©^2024, Wiley–VCH GmbH)

Zhang et al. [146] engineered a 3D-printed CoSi/PCL composite scaffold exhibiting NIR-II photothermal capabilities and improved adipogenic activity for post-mastectomy breast reconstruction. This composite integrated polycaprolactone (PCL) with cobalt orthosilicate (Co_2_SiO_4_, CoSi), yielding a multifunctional scaffold. The photothermal properties enabled effective tumor ablation under light irradiation, while the release of silicate and cobalt ions promoted adipogenesis and angiogenesis, facilitating scaffold integration with surrounding tissues. The combination of tumor treatment and breast reconstruction marks a promising direction in breast cancer therapy. Recently, Wang et al. [147] developed a 3D-printed prosthesis made of therapeutic hydrogel. The newly designed prosthesis not only reconstructed the breast but also smartly sensed tumors and inhibited tumor recurrence. (Fig. 11a). The study designed three candidate prostheses with numerous pores and different diameters of internal columns (Fig. 11b). To select the most suitable prosthesis, the compressive modulus test and cyclic compression test were performed. The results showed that prosthesis-A exhibited a compressive modulus closest to that of two commercial prostheses (Fig. 11c), suggesting that prosthesis-A, with a 1.0 mm diameter of the internal column, could be a good candidate for breast reconstruction. Meanwhile, this hydrogel exerted an anti-tumor effect by loading RSL3 and enhancing ROS in TME to trigger ferroptosis in tumor cells. In the 4T1-Luc mouse model, a single intratumoral implantation of the RSL3@LIPO@GEL resulted in a rapid decrease from day 10 to day 30 (Fig. 11d), and the tumor volume curve was significantly lower than that of the empty gel and liposome-alone groups, suggesting that the ROS-triggered sustained drug release strategy showed an outstanding tumor inhibition effect (Fig. 11e). Importantly, the body weight of mice in the treatment group remained stable throughout the observation period, and no signs of systemic toxicity were observed, providing a good safety basis for subsequent clinical translation (Fig. 11f).

### Hydrogel-based tumor-research model construction

Hydrogels, with their 3D architectures akin to the native ECM and their tunable physicochemical properties, have emerged as the material of choice for engineering biomimetic TME and organoid culture systems. Currently, research on hydrogels mainly focuses on two aspects: extracellular matrix simulation and the construction of tumor-targeted organoids.

Hydrogels have emerged as valuable tools for modeling microenvironmental cues that govern BC metastasis, including matrix stiffness, porosity, and biochemical factor gradients (e.g., VEGF, TGF-β). These biomimetic platforms enable the investigation of tumor-stromal cell interactions, drug resistance evolution, and immune escape mechanisms. To elucidate the collective traversal of the physiological basement membrane (BM) during BC progression, Chaudhuri et al. [148] developed a three-dimensional in vitro model of collective invasion of the BM during breast cancer. The results showed that cells utilized both proteases and forces, but not invadopodia-to breach the BM. Forces were generated by a combination of global cell volume expansion, which stretched the BM, and local contractile forces acting in the plane of the BM to breach it, allowing invasion. These results uncovered a mechanism by which cells collectively interact to overcome a critical barrier to metastasis.

Organoids play a vital role in predicting drug responses, guiding clinical treatment regimens, and elucidating the mechanisms of tumor metastasis. Tumor-derived organoids, developed by combining patient-derived tumor tissues or circulating tumor cells with hydrogel scaffolds, can recapitulate the heterogeneity and biology of the original tumor. These models not only mimic TME but also provide substantial support for personalized therapy. Hydrogel-based organoid nanomaterials are transitioning from basic research to preclinical studies, demonstrating great translational potential. Patient-derived tumor organoids (PDOs) are particularly promising preclinical models, as they faithfully recapitulate the histology, gene expression, and drug response profiles of the donor patient's tumor. Prince et al. [149] developed a nanofibrillar hydrogel (EKGel) to initiate and promote the growth of breast cancer PDOs. PDOs cultured in EKGel exhibited histopathological features, gene expression profiles, and drug responses compared with parental tumors and basement membrane extract (BME). Moreover, EKGel demonstrated reduced batch-to-batch variability, a spectrum of mechanical properties, and minimized contamination from murine cells. These findings suggested that EKGel was a superior alternative to BME matrices for the initiation, growth, and maintenance of breast cancer PDOs.

The revolutionary potential of hydrogels in the biomedical sector is continuously expanding and evolving as research on these remarkable materials becomes increasingly thorough and sophisticated. Hydrogels have demonstrated exceptional promise in a variety of critical areas, including, but not limited to, drug delivery, 3D cell biology, wound healing, regenerative medicine, and the development of biosensors that can monitor health conditions in real time [150].

### Patents survey and clinical trials

#### Patents survey

Nanoscale hydrogels, characterized by tunable viscoelasticity, high water content, and stimuli-responsive swelling, have been extensively exploited for BC applications, including controlled drug delivery, post-surgical tissue reconstruction, and the construction of multimodality imaging vectors. To date, an increasing number of patents based on hydrogel-based nanomaterials for BC therapy have been granted (Table 5).

**Table 5:** Related patents of hydrogel-based nanoparticles in breast cancer

Patent number	Publishing date	Hydrogel-based nanoparticles	Function	Ref
CN106237336A	2016–12–21	EPI@PTX@GDL gel	Drug delivery	[151]
CN110038025A	2019–07–23	RNA triple helix hydrogel	Targeted therapy of TNBC	[152]
US11155830B2	2021–10–26	DOX/MnO_2_@Ce6 gel	Drug delivery	[153]
CN114939129A	2022–08–26	Acellular matrix composite hydrogel	Drug delivery	[154]
CN116098857A	2023–05–12	DTX/USIO@PEI NGs/siCD47	Drug delivery	[155]
CN116725944A	2023–09–12	Fe_3_O_4_-CS-GelMA	Drug delivery; Adipose tissue defect repair	[156]
CN116763907A	2023–09–19	IM-CS-NPs/E7/ATP@ALG	Drug delivery	[157]
CN116966134A	2023–10–31	SHK/AuNR@gel	Drug delivery	[158]
CN118286473A	2024–07-05	^131^IAu-coated DA/GEM hydrogel	Wound healing; Drug delivery	[159]
CN118374451A	2024–07–23	EGF/Ca^2+^@ALG hydrogel	Tissue engineering	[160]
CN118772233A	2024–10–15	Polypeptide hydrogel	Targeted therapy	[161]
CN119112760A	2024–12–13	TMP@PEG gel	Drug delivery	[162]
WO2025051264A1	2025–03–13	Multi-layer organization	In-situ tumor modeling	[163]
CN119700648A	2025–03–28	PE NPs/HAase@ALG hydrogel	Drug delivery	[164]
WO2025139283A1	2025–07-03	Chitosan/HA/M@AuNPs	Radiosensitization	[165]

Early patent technology for hydrogels primarily focused on facilitating localized, sustained release of chemotherapy agents, such as DOX and PTX. The core value resides in minimizing systemic toxicity while enhancing local drug concentration. Recently, advancements in materials science have prompted a shift in hydrogel research from "passive drug delivery" to "active modulation of the tumor immune microenvironment". Furthermore, recent progress has extended to tissue repair following breast cancer surgery, emphasizing the construction of hydrogel scaffolds that serve dual purposes: preventing tumor recurrence and guiding soft tissue regeneration. This strategy aims to restore tissue morphology and function while ensuring complete tumor eradication.

#### Clinical trials

Currently, the application of hydrogels in breast cancer has evolved from managing side effects to playing an active role in diagnosis and treatment (Table 6). Early research focused on leveraging their physical properties to mitigate adverse reactions, such as in clinical trials to prevent radiation dermatitis.

**Table 6:** Current clinical trials involving hydrogel-based nanomaterials systems in breast cancer

NCT number	Drug	Indications	Phase
NCT04899908	Aguix Gadolinium-Based Nanoparticles	Brain Metastases	Phase 2
NCT06169072	superparamagnetic iron oxide nanoparticles (SPIO)	Sentinel Lymph Node Localisation	Phase 2
NCT04951245	Carbon nanoparticle suspensions (CNSs) plus ICG	Sentinel Lymph Node Biopsy (UltraCars)	Phase 3
NCT05625698	SPIO Magtrace^®^	Premarking of axillary nodes	Not Applicable
NCT05079763	Bacterial cellulose-monolaurin hydrogel	Acute radiation dermatitis	Phase 2
NCT02839473	Hydrogel Hydrosorb^®^	Radio-induced skin toxicity	Phase 3
NCT00481884	RadiaPlexRx Hydrogel	Radiation dermatitis	Phase 3
NCT05800834	MorphineGEL	Pain reduction in patients with cancer wounds	Phase 2
NCT05073172	Silicone-based film forming topical gel (StrataXRT)	Radiation dermatitis in BC or head and neck cancer patients	Not Applicable
NCT04995328	Radiation Care^®^ gel	Radiation-Induced Dermatitis	Not Applicable
NCT04481802	RadiaAce Gel	Radiation dermatitis	Not Applicable
NCT06860815	Y-90 SIR-S Spheres	Liver directed metastatic BC	Phase 2
-	RadioGel^®^[167]	Solid cancerous tumors of the head and neck	The initial clinical trial has been completed

Data are from the website https://clinicaltrials.gov/. “-” represents unknown

Currently, the application of hydrogels in breast cancer has evolved from managing side effects to playing an active role in diagnosis and treatment (Table 6). Early research focused on leveraging their physical properties to mitigate adverse reactions, such as in clinical trials to prevent radiation dermatitis. With advancements in materials science, the application of hydrogels has expanded to critical aspects of tumor diagnosis and treatment. For example, superparamagnetic iron oxide nanoparticles (SPIO, Magtrace^®^) and carbon nanosuspensions (CNSs) were frequently employed for sentinel lymph node tracing, offering a reliable method for precise lymph node staging during surgery. The BioZorb^®^ absorbable hydrogel, developed by Hologic, has been implemented in clinical practice [166]. This innovative product integrates a three-dimensional scaffold with titanium markers. It served the dual purpose of accurately directing post-breast-conserving surgery radiotherapy and facilitating local tissue healing and restructuring. Consequently, it has emerged as a pivotal surgical adjunct in practical clinical applications.

Additionally, certain functionalized nanoparticles have begun to enhance the efficacy of radiotherapy or facilitate localized treatment. Gadolinium-based nanoparticles were used for radiotherapy sensitization of brain metastases, while yttrium-90 resin microspheres were used for directional internal irradiation therapy for liver metastases. Notably, yttrium-90 microsphere radioembolization has evolved into a well-established local treatment modality in clinical practice and has received global approval for the treatment of unresectable hepatocellular carcinoma (HCC) and liver metastases from colorectal cancer. The treatment of liver metastases originating from breast cancer is also highly anticipated. Notably, Vivos Inc. has recently announced the initiation of the first human clinical trial of RadioGel^®^ precision radionuclide therapy^™^ in India. Five patients with lymph node cancer successfully underwent treatment in the RadioGel^®^ trials, marking a significant advancement in novel cancer therapies. Through the dual analysis of patent and clinical trial data, we found that hydrogel-based nanomaterial systems have the potential to transform breast cancer treatment beyond the theoretical level. Although this achievement represents a critical step forward in the clinical application of nanomaterials, substantial challenges remain for the clinical implementation of hydrogel-based nanomaterials.

## Conclusions and Perspectives

### Conclusions

Hydrogels represent a transformative platform in BC therapy, bridging materials science and clinical oncology. This review systematically examines the progress of hydrogel-based nanomaterials in BC, covering precision drug delivery, stimuli-responsive therapeutic activation, drug administration, immune modulation, application, and clinical translation of hydrogels. By integrating chemotherapy agents, targeted therapeutics, and TCM compounds into adaptable hydrogel matrices, researchers have achieved controlled drug release, enhanced bioavailability, and reduced systemic toxicity. The exploration of collaborative therapies, combining dual- or multiple-stimulus-responsive mechanisms with multimodal administration routes, has unlocked synergistic antitumor efficacy, particularly in the treatment of metastatic diseases and radioresistance. Notably, the immune-modulatory function of hydrogels, including modulation of macrophage polarization and activation of cytotoxic T cells, positions them as pivotal tools for developing next-generation immuno-oncology strategies to enhance the body's natural cancer-fighting ability.

Hydrogel-based nanomaterials continue to reshape the breast cancer therapeutic landscape, driven by ongoing advancements in material chemistry and structural design. Preclinical studies have demonstrated their versatile functions, from scavenging ROS after radiotherapy to orchestrating immune-cell recruitment and presenting tumor antigens in a single injectable depot. As formulation strategies evolve toward long-term stability, on-demand degradability, and scalable GMP-compliant production, these hydrogel platforms show strong potential to transition from the laboratory to clinical settings. Their further development may eventually transform post-resection management and contribute to a more integrated approach in oncology.

### Perspectives

Hydrogels, versatile and biocompatible platforms, have demonstrated immense potential to revolutionize breast cancer therapy, particularly through the development of intelligent diagnostic systems and multimodal treatment strategies. Preliminary research highlighted promising avenues, including AI-driven hydrogels, closed-loop therapeutic platforms, and immune-metabolic reprogramming strategies, all aimed at achieving precise, adaptive, and synergistic anti-tumor effects. These systems leverage the unique properties of hydrogels, including tunable physicochemical characteristics, high water content, and the ability to encapsulate and controllably release therapeutic agents, to address the complexities of the TME.

Despite this promise, the journey from laboratory innovation to the clinical translation of hydrogel-based nanomaterials is fraught with a series of multidimensional bottlenecks spanning material science, biology, engineering, and regulation. The primary challenges can be systematically categorized as follows:

1. Material design and stability: At the foundational level, ensuring uniform dispersion and long-term stability of functional nanoparticles (e.g., drugs, imaging agents, or stimuli-responsive elements) within the hydrophilic hydrogel network is paramount. Preventing nanoparticle aggregation and premature leakage at non-target sites is crucial for achieving the intended therapeutic efficacy and minimizing off-target effects.
2. Biosafety and nanotoxicology: Clinical applications require a thorough, nuanced understanding of the biosafety profiles of the constituent nanomaterials. Potential toxicity may arise from various sources, including physical damage, chemical reactivity, immunogenicity, and challenges associated with long-term retention and metabolic clearance. Key material properties-such as size, morphology, surface charge, chemistry, aggregation state, dosage, and exposure routes-profoundly influence these biological interactions and associated risks. Advancing safety assessment requires not only standard in vitro and in vivo evaluations but also: (i) a deeper exploration of the physicochemical properties governing nanomaterial-biology interactions; (ii) the establishment of multi-tiered evaluation models; (iii) the development of high-sensitivity, high-resolution techniques for biological effect studies; and (iv) the integration of computational toxicology and AI-powered predictive models for prospective safety screening.
3. Scalable manufacturing and quality control: Transitioning from lab-scale synthesis to Good Manufacturing Practice (GMP)-compliant large-scale production poses significant engineering challenges. Consistently replicating critical parameters-such as nanoparticle dispersion homogeneity, hydrogel mechanical properties, and structural integrity post-sterilization-across different production batches is essential for clinical viability. Furthermore, the standardization of hydrogel degradation kinetics, which critically impacts drug release profiles and in vivo residence time, remains an area requiring extensive investigation.
4. Clinical efficacy and the translational gap: A significant disconnect often exists between therapeutic outcomes in animal models and human patients. For instance, phenomena like the enhanced permeability and retention (EPR) effect, which is pronounced in many murine tumor models, exhibit high heterogeneity and limited reliability in human cancers. This gap underscores the need for more predictive preclinical models.
5. Regulatory pathway for combination products: Hydrogel-based therapeutic systems typically fall under the regulatory category of “combination products,” involving both a device (the hydrogel matrix) and a drug or biologic component. Regulatory agencies exercise considerable caution in evaluating such complex products, requiring exhaustive data on safety, efficacy, and quality control. The approval pathway for these innovative systems remains relatively immature and fraught with uncertainty, posing a substantial barrier to clinical entry.

Looking forward to bridging the gap between promising preclinical research and successful clinical deployment, future efforts should be strategically directed toward the following key areas:

- (1) Next-generation material design: Focus on developing “smart” hydrogel systems with enhanced stability, precisely engineered degradation profiles, and improved capacity for spatiotemporally controlled release. Innovations should aim to mitigate nanoparticle leakage and aggregation while ensuring responsive behavior within the dynamic TME.
- (2) Advanced biosafety and predictive toxicology: Prioritize the creation of comprehensive, standardized datasets on the biological interactions of hydrogel nanomaterials. This will be instrumental in developing robust machine learning and computational models that can predict, explain, and guide the design of safer materials. Investing in high-resolution analytical techniques to study bio-nano interactions at the molecular level will be crucial.
- (3) Translation-oriented engineering and standardization: Research must increasingly address scalable fabrication processes, reliable sterilization methods that preserve hydrogel functionality, and the establishment of standardized protocols for characterizing critical quality attributes (e.g., degradation kinetics, drug release profiles). Collaboration with engineering and regulatory science experts early in the development process is essential.
- (4) Elucidation of complex biological mechanisms: A deeper fundamental understanding of the intricate crosstalk between immune and metabolic pathways in tumors is needed to design more effective combination therapies. Furthermore, research must focus on validating therapeutic mechanisms and controlled-release triggers in clinically relevant models to predict human outcomes better.
- (5) Navigating the regulatory landscape: Proactive engagement with regulatory bodies to define clear development pathways for combination products is necessary. Generating high-quality, comprehensive data packages tailored to regulatory expectations will be key to de-risking the clinical translation process.

In conclusion, while hydrogel-based intelligent therapeutic systems represent a frontier in precision oncology, their clinical translation demands a concerted, interdisciplinary effort. By systematically addressing the intertwined challenges of material design, safety, manufacturing, biological efficacy, and regulation, we can accelerate the development of robust, effective, and clinically viable hydrogel nanotechnologies, ultimately transforming the landscape of cancer treatment.

## Data Availability

No datasets were generated or analysed during the current study.

## Declarations

### Declarations

Scheme 1, Figs. 4, 5, and 10 of this manuscript were created in https://BioRender.com.
